# Supplementary material for: A unified European hydrogen infrastructure planning to support the rapid scale-up of hydrogen production
Source: Nat Commun. 2024 Jun 29;15:5517. doi: 10.1038/s41467-024-49867-w (PMC11217447; doi:10.1038/s41467-024-49867-w)
Supplement: Supplementary file 1 — Supplementary Information [file 41467_2024_49867_MOESM1_ESM.pdf]

Supplementary Information: A unified European hydrogen infrastructure planning to support the rapid scale-up of hydrogen production

Ioannis Kountouris[1*], Rasmus Bramstoft[1], Theis Madsen[1], Juan Gea-Bermúdez[2], Marie Münster[1], Dogan Keles[1]

[1]Department of Technology, Management and Economics, Technical University of Denmark, Produktionstorvet, Bygning 424, Kongens Lyngby, 2800, Denmark.
[2]Joint Research Centre (JRC), European Commission, Calle Inca Garcilaso, 3, Sevilla, 41092, Spain.

*Corresponding author(s). E-mail(s): iokoun@dtu.dk;
Contributing authors: rabpe@dtu.dk; tmad@dtu.dk; juan.gea-bermudez@ec.europa.eu; maem@dtu.dk; dogke@dtu.dk;

Contents

Supplementary Method 1 3

Supplementary Method 2 5

Supplementary Method 3 5

Supplementary Method 4 7

Supplementary Method 5 8

Supplementary Method 6 11

Supplementary Method 7 12

Supplementary Method 8 14

Supplementary Method 9 16

Supplementary Method 10 19

Supplementary Method 11 26

Supplementary Method 12 27

Supplementary Figures 32

Supplementary Discussion 1 38

Supplementary Note 1 40

Supplementary Note 2 40

Supplementary Note 3 43

Supplementary Note 4 45

Supplementary Note 5 49

Supplementary Note 6 53

Supplementary Note 7 53

Supplementary Note 8 54

Supplementary References 57

Supplementary Method 1

Hydrogen mathematical modelling formulation in Balmorel

The objective of Balmorel is to minimize the total energy system cost. The objective function is subject to constraints for the entire energy system responsible for generating, storing, transmitting, and converting multiple vectors such as electricity, heat, gas, and others. Here, we present a simplified version of the main mathematical formulations related to hydrogen modeling due to the focus of this paper. All parameter names are denoted in small letters, while all variable names are in capital. All equations in Balmorel are available in the latest documentation [1] or can be found in the available source code (see section Data and Code availability, main article).

To simulate the future European energy system, we adopt a myopic modeling approach, simulating the period between 2020 and 2050, by splitting in 5 years intervals. In addition, every year is subdivided into seasons $s \in S$, which are in total 52, and every s is further divided into 168 time segments $t \in \mathcal{T}$. Due to computational efficiency and tractability, a reduced amount of time steps and seasons are carefully selected by applying the methodology described in ref. [2].

Equation 1 represents the objective function and, as a result of the optimization, the total annual cost for satisfying the electricity, heating, and hydrogen demands. The variable costs of operation of the technology g, including costs related to fuel consumption and environmental taxes, are given by c_g^{VOP}. The amount of commodity associated with those variable costs, produced or consumed by a specific technology g, located in the area a, in year y at each time period, defined by the season s, temporal slice t, is given by $P_{a,s,t,g}$. The capital expenditures in technology g are defined as a product of the technology costs c_g^{CAP} by the variable $P_{a,g}^{new}$, which represents the capacity installed of technology g in area a. To express the investment and operational expenditures as annual costs, we discount the total costs based on an annuity factor $f = (1-(1+\tau)^{-n})/\tau$, assuming a social discount rate τ of 4 % [3] and considering technology f_g or transmission f_r specific lifetime.

$$\begin{aligned} \text{Min} \sum_{\substack{a\in\mathcal{A}\\ s\in\mathcal{S}\\ t\in\mathcal{T}\\ g\in\mathcal{G}}} f_g c_g^{VOP} P_{a,s,t,g} + \sum_{\substack{a\in\mathcal{A}\\ g\in\mathcal{G}}} f_g c_g^{FOP}(p_{a,g}^{ex} + P_{a,g}^{new}) + \sum_{\substack{a\in\mathcal{A}\\ g\in\mathcal{G}}} f_g c_g^{CAP} P_{a,g}^{new} \\ + \sum_{r,r'\in\mathcal{R}_{r,r'}^{ex}} f_r c_{r,r'}^{CAP} P_{r,r'}^{trnew} \end{aligned} \quad (1)$$

Equation 2 ensures that the hydrogen demand, $d_{r,s,t}^{H_2}$, is met in all regions (geographical areas a are aggregated into transmission regions r and time periods. The demand is split into two main categories, endogenous and exogenous. Endogenous demand could appear due to the need for hydrogen to power or to produce biomethane. Exogenous demands are related to hydrogen penetrating the transport sector, producing liquid fuels and chemicals, or directly being used for high-value heat in the industry. Hydrogen might be transmitted between regions. $P_{r,r',s,t}^{trans}$ shows the amount

of hydrogen exported from region $r \in \mathcal{R}$ to a connected region $r' \in \mathcal{R}^{exp}_{r,r'}$, and the variable $P^{trans}_{r',r,t}$ denotes the amount of hydrogen imported, including losses $e_{r',r}$, from the region $r' \in \mathcal{R}^{imp}_{r',r}$ towards r during the time segment (s,t). Furthermore, hydrogen storage can be invested in and utilized to balance hydrogen production and demand. Every storage can load $p^{H_2,toSTO}_{a,g,s,t}$ and unload $P^{H_2,fromSTO}_{a,g,s,t}$ accordingly.

$$\begin{aligned}&\sum_{\substack{a\in\mathcal{A}^R_r\\ g\in\mathcal{G}^{H2}}} P^{H_2}_{a,g,s,t} + \sum_{r'\in\mathcal{R}^{im}_{r',r}} e_{r',r}P^{H_2,trans}_{r',r,s,t} - \sum_{r'\in\mathcal{R}^{ex}_{r,r'}} P^{H_2,trans}_{r,r',s,t}\\ &-\sum_{\substack{a\in\mathcal{A}^R_r\\ g\in\mathcal{G}^{H_2,STO}}} P^{H_2,toSTO}_{a,g,s,t} + \sum_{\substack{a\in\mathcal{A}^R_r\\ g\in\mathcal{G}^{H_2,St}}} P^{H_2,fromSTO}_{a,g,s,t} = d^{H_2}_{r,s,t}, \quad \forall r\in\mathcal{R}, s\in\mathcal{S}, t\in\mathcal{T}\end{aligned} \tag{2}$$

Equation 3 represents the dynamic equation for hydrogen storage modeling. In Balmorel, storage technologies are categorized as short-term or seasonal storage, yet the two types are handled differently. For more information, see [1]. Hydrogen storage is modeled as seasonal. The hydrogen storage content $V^{H_2,STO}_{a,g,(s+1,t)}$ at the next season s and $V^{H_2,STO}_{a,g,(s,t+1)}$ at the next time step t, is equal to the hydrogen storage content, $V^{H_2,STO}_{a,g,s,t}$, at the beginning of the time segment (s,t), plus the difference between loading the hydrogen storage, $P^{H_2,toSTO}_{a,g,s,t}$, and unloading, $P^{H_2,fromSTO}_{a,g,s,t}$, while also considering the efficiency loss, $\epsilon^{H_2,STO,eff}_g$. The difference is multiplied by the length of the chronological time segment, $\gamma_{s,t}$. Furthermore, due to time aggregation, an additional term δ_s representing the number of seasons aggregated into one season s is introduced to capture the inter-seasonal variability. Essentially the equations 3 and 4 model both a seasonal and hourly inter-temporal balance of the hydrogen storage technologies.

$$\begin{aligned}V^{H_2}_{a,g,(s,t+1)} = +V^{H_2}_{a,g,s,t} + \gamma_{s,t}P^{H_2,toSTO}_{a,g,s,t} - \gamma_{s,t}\frac{P^{H_2,fromSTO}_{a,g,s,t}}{\epsilon^{H_2,eff}_g},\\ \forall a\in\mathcal{A}, g\in\mathcal{G}^{H_2,STO}, s\in\mathcal{S}, t\in(\mathcal{T}-1)\end{aligned} \tag{3}$$

$$\begin{aligned}&V^{H_2}_{a,g,(s,t+1)} + V^{H_2}_{a,g,(s+1,t')} = (\delta_s-1)\left(v^{H_2}_{a,g,s} + \gamma_{s,t}\left(p^{H_2,toSTO}_{a,g,s,t} - \frac{p^{H_2,fromSTO}_{a,g,s,t}}{\epsilon^{H_2,eff}_g}\right) - v^{H_2}_{a,g,s,t'}\right)\\ &+V^{H_2}_{a,g,s,t} + \gamma_{s,t}P^{H_2,toSTO}_{a,g,s,t} - \gamma_{s,t}\frac{P^{H_2,fromSTO}_{a,g,s,t}}{\epsilon^{H_2,eff}_g} \quad a\in\mathcal{A}, g\in\mathcal{G}^{H_2,STO}, \quad \forall s\in\mathcal{S}, t\in\mathcal{T}, t'=1\end{aligned} \tag{4}$$

Equation 5 describes the limits to hydrogen transmission $P^{trans}_{r,r',s,t}$ between interconnected regions $r-r'$ given by the capacity of the existing lines, $p^{trex}_{r,r'}$ and new $P^{trnew}_{r,r'}$, and their availability $k^{tr}_{r,r',s,t} \in [0,1]$ at the time segment (s,t). Furthermore, the expansion of the hydrogen grid $P^{trnew}_{r,r'}$ is associated with corresponding costs as described in the methods section.

$$P^{trans}_{r,r',s,t} \leq k^{tr}_{r,r',s,t}(p^{trex}_{r,r'} + P^{trnew}_{r,r'}), \forall\ r, r' \in \mathcal{R}^{ex}_{r,r'}, t \in \mathcal{T} \tag{5}$$

Supplementary Method 2

Flexible spatial hydrogen derivative fuel demand Balmorel is a partial equilibrium model that satisfy exogenous energy demand, which often are spatially fixed per region r for electricity and hydrogen demands. To adequately examine the developed hydrogen network topology and sizing, the uncertainty related to spatial locations of synthetic fuel demand in the future can be examined by partially endogenizing the hydrogen derivatives production locations across the Pan-European energy system. To do so, the nodal hydrogen balance Equation 2 is expanded to Equation 6 accounting for direct hydrogen demand $d^{H_2-direct}_{r,s,t}$ and hydrogen demand for synthetic fuels production $D^{SynFuels}_{r,s,t}$ corresponding to high-value chemicals and ammonia (see Supplementary Method 9). A parameter $\gamma \in [0, 1]$ determines the potential shift in production locations relative to the exogenous regional demand of hydrogen for synthetic fuel production $d^{SynExog}_{r}$. Equation 7 and 8 constraints the new spatially allocated (or endogenously optimized) demand level ($V^{SynShift}_{r}$) providing an upper and lower new level per region r. The new derivatives demand is down-scaled to seasons s and time segments t based on an exogenously defined profile $p^{profile-SynFuels}_{r,s,t}$. Lastly, Equation 10 maintains an overall balance between the original system-level demand and the final shifted derivatives fuel demand.

$$\sum_{\substack{a\in\mathcal{A}^R_r\\ g\in\mathcal{G}^{H2}}} P^{H_2}_{a,g,s,t} + \sum_{r'\in\mathcal{R}^{im}_{r',r}} e_{r',r} P^{H_2,trans}_{r',r,s,t} - \sum_{r'\in\mathcal{R}^{ex}_{r,r'}} P^{H_2,trans}_{r,r',s,t} - \sum_{\substack{a\in\mathcal{A}^R_r\\ g\in\mathcal{G}^{H_2,STO}}} P^{H_2,toSTO}_{a,g,s,t} + \sum_{\substack{a\in\mathcal{A}^R_r\\ g\in\mathcal{G}^{H_2,St}}} P^{H_2,fromSTO}_{a,g,s,t} = d^{H_2-direct}_{r,s,t} + D^{SynFuels}_{r,s,t}, \quad \forall r \in \mathcal{R}, s \in \mathcal{S}, t \in \mathcal{T} \tag{6}$$

$$(1-\gamma) d^{SynExog}_{r} \leq V^{SynShift}_{r}, \quad \forall r \in \mathcal{R} \tag{7}$$

$$V^{SynShift}_{r} \leq (1+\gamma) d^{SynExog}_{r}, \quad \forall r \in \mathcal{R} \tag{8}$$

$$D^{Synfuels}_{r,s,t} = V^{SynShift}_{r} p^{profile-SynFuels}_{r,s,t}, \quad \forall r \in \mathcal{R}, s \in \mathcal{S}, t \in \mathcal{T} \tag{9}$$

$$\sum_{r} V^{SynShift}_{r} = \sum_{r} d^{SynExog}_{r} \tag{10}$$

Supplementary Method 3

Myopic, limited and perfect foresight - differences

This study employs a myopic optimization technique to tackle seven consecutive optimization problems (2020-2050, by 5-year intervals) in sequential order. The problems are connected through the use of the optimized results from the previous problem as input data for the subsequent problem. Another approach is to utilize perfect foresight, where all years are optimized simultaneously assuming perfect information, resulting in an overall lower total system cost. An intermediate approach capable of varying degrees of foresight may result from the application of limited foresight via a rolling horizon. The Balmorel model supports all of the three techniques mentioned. While myopic optimization may lead to reduced problem size and, consequently, a shorter computation time when compared to perfect foresight, the variations in the levels of information available may cause variations in the pathway-optimized decisions across the two techniques. We try to quantify the robustness of our results in regard to the lock-in effect of blue hydrogen by applying those three techniques.

The model is solved on a High-Performance Computing (HPC) server utilizing 2x Intel Xeon Gold 6342 (24 core, 2.8 GHz) and 512GB memory (16 x 32GB DDR4-3200) [4]. Due to the problem's complexity, 2020-2050 with 5-year intervals, perfect foresight was not able to reach a feasible solution. Therefore, we decreased the problem size to examine the foresight effects, simulating 2020-2050 with a 10-year interval.

Supplementary Fig. 1 depicts that independent of the chosen model foresight technique, the lock-in effect is projected and captured under the current data and system assumptions. Our findings are supported by the literature. A recent study by Lambert et al. (2023) [5] highlights that although the final European energy system looks almost the same across the two methodologies, there could be deviations in the intermediate years. However, another study by Siala et al. (2022) [6] compares optimal generation expansion of a European energy system results for five different models assuming either myopic or perfect foresight. Similar to our analysis, they demonstrate that under a high CO_2 price scenario, the result differences are not significant. Furthermore, we highlight that the computational time dramatically decreases when shifting from perfect to myopic foresight. For instance, the myopic solution required 22 hours of solution time. The perfect foresight resulted in a simulation run of more than 364 hours. Babrowski et al. (2014) [7] noted that the myopic approach with stable input parameters is just as applicable as the perfect foresight approach, with the added advantage of requiring significantly less computing time.

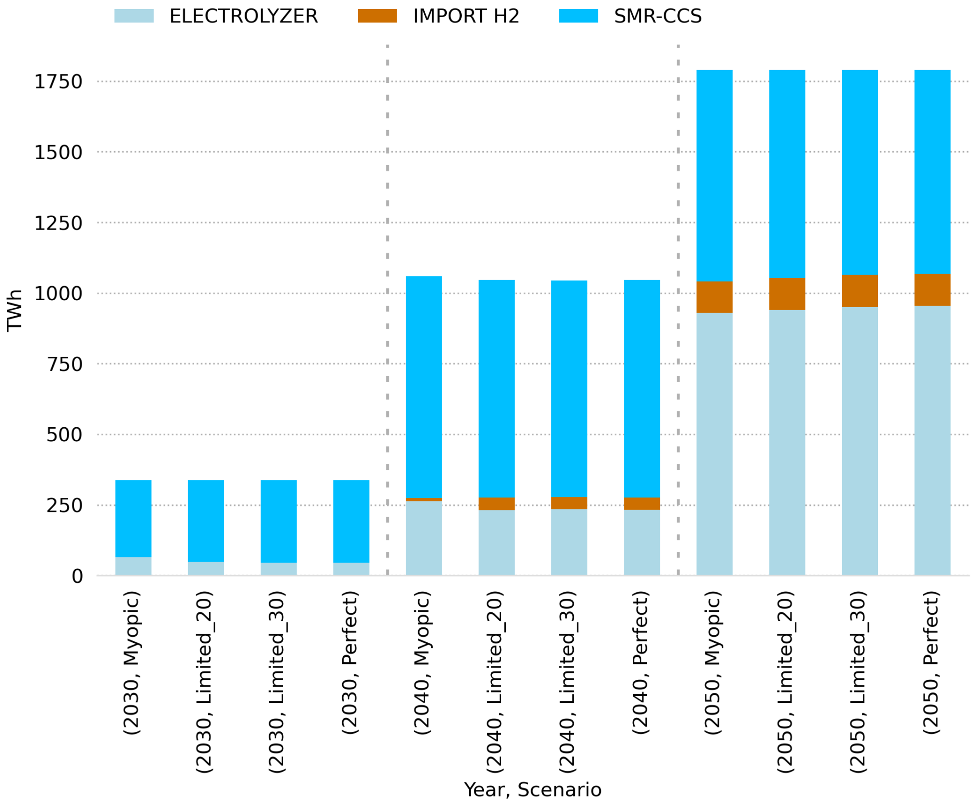

Supplementary Fig. 1: Sensitivity analysis of Hydrogen Europe (H2E) scenario hydrogen production pathway. Varying the foresight information. Myopic, Limited foresight with optimizing 20 years together, Limited foresight optimizing 30 years together, Perfect.

Supplementary Method 4

Green hydrogen and RFNBOs

With the latest delegated acts for the production of renewable liquid and gaseous transport fuels of non-biological origin (RFNBO), the European Commission is establishing detailed rules and requirements for classifying hydrogen produced by water electrolysis as "renewable" or so-called "green" [8]. This study has no direct restriction that electrolytic hydrogen must be produced with renewable electricity or strictly comply with the latest EU requirements. We observe that the implemented CO_2 quota price trajectory is steep (see Supplementary Note 6), thereby accelerating the power system's transition to renewable and low-carbon electricity generation before 2035. In addition, we investigate the hourly electricity generation mix and demonstrate

that hydrogen production follows the availability of renewable energy sources (Supplementary Fig. 2). Even though natural gas based conventional power plants without carbon capture and storage (CCS) remain in the system through 2030, a negative temporal correlation is observed between electrolytic hydrogen production and carbon-intensive electricity generation. We do not observe a geographical (i.e., regional) correlation when electrolytic hydrogen production occurs alongside conventional peak load services.

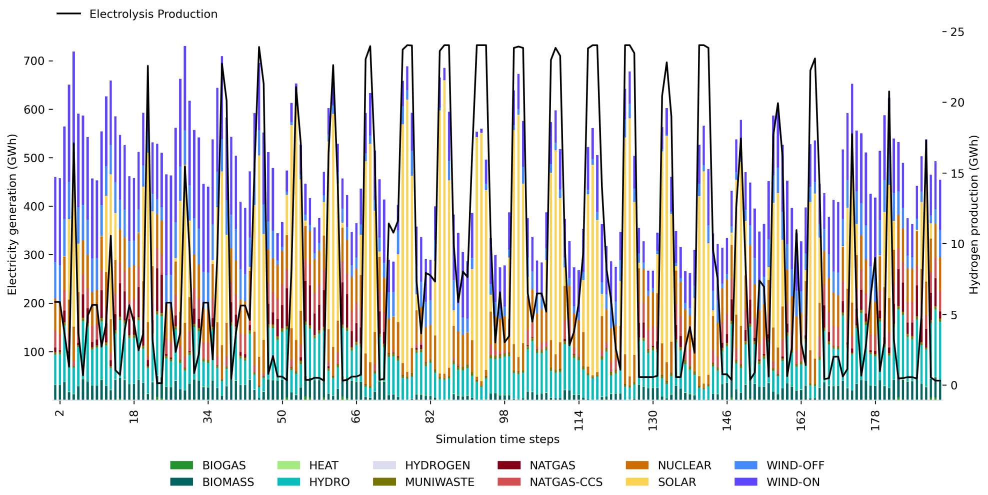

Supplementary Fig. 2: Electricity mix, Hydrogen Europe (H2E) scenario. Left y-axis aggregated hourly electricity generation mix for EU 27, the United Kingdom, Norway, Switzerland, and the remaining Balkan nations, by 2030. The right y-axis presents the total hydrogen production. The black line depicts the optimal dispatching of water electrolysis units.

Supplementary Method 5

Network expansion: Hydrogen

This study expands the geographical scope of Balmorel to encompass a total of 35 countries. The model supports a three-layer spatial resolution of countries, regions, and areas (for more information, see. ref. [1]). The power and hydrogen networks are developed and optimized at a regional resolution. The European model is divided into 46 regions in total. We assume a one-node region representation for the majority of the countries, but in other cases, such as the Nordics, we use the Nordpool bidding zone regional split.

In the main manuscript, see section Hydrogen infrastructure, network expansion and storage, we provided a method for classifying future hydrogen grid connections

according to their characteristics (new, repurposed, onshore, and offshore). The associated costs are extracted from the most recent European Hydrogen Backbone (EHB) report [9]. For the intermediate year of 2035, the hydrogen grid costs are linearly interpolated. The EHB report does not provide updated cost projections beyond 2040. We presume that the cost of hydrogen transmission will not change after 2040 and in subsequent years. Supplementary Table 1 summarizes an overview of the costs. Supplementary Fig. 3a and 3b illustrate the final cross-regional hydrogen network expansion expenditure costs. Lastly, similar to the electricity network development (see Supplementary Method 7), the hydrogen network is built by assuming linear bi-directional flow.

There are additional operational costs for delivering hydrogen via transmission pipelines. Energy expenses for compression are implicitly accounted for by reducing the overall efficiency of the electrolyzer units by 2.1 %, 1.7 % and 1.5 % for investments made in 2030, 2040, and 2050, respectively. Furthermore, to maintain the operational pressure, hydrogen transmission energy losses of 0.0022 %/km [3] are included. A 4 % discount rate and a lifetime expectancy of 50 years is assumed for the network infrastructure investments. Other costs associated with hydrogen distribution grids and the necessary equipment to supply hydrogen to the consumption sites are not considered.

Supplementary Table: 1: Hydrogen network expansion costs for 2030-2040.

Year	Onshore new (€/MW/km)	Onshore repurposed (€/MW/km)	Offshore new (€/MW/km)	Offshore repurposed (€/MW/km)
2030	536.17	150.00	902.13	175.00
2035	399.63	118.08	676.45	145.50
2040	263.08	86.15	450.77	120.00

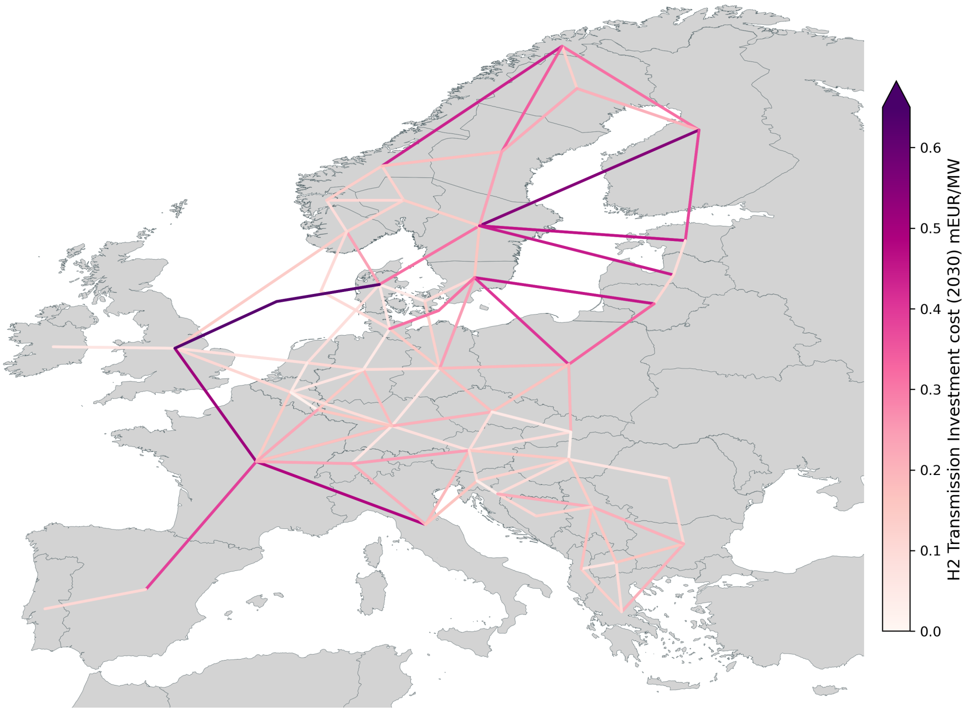

(a) Year:2030

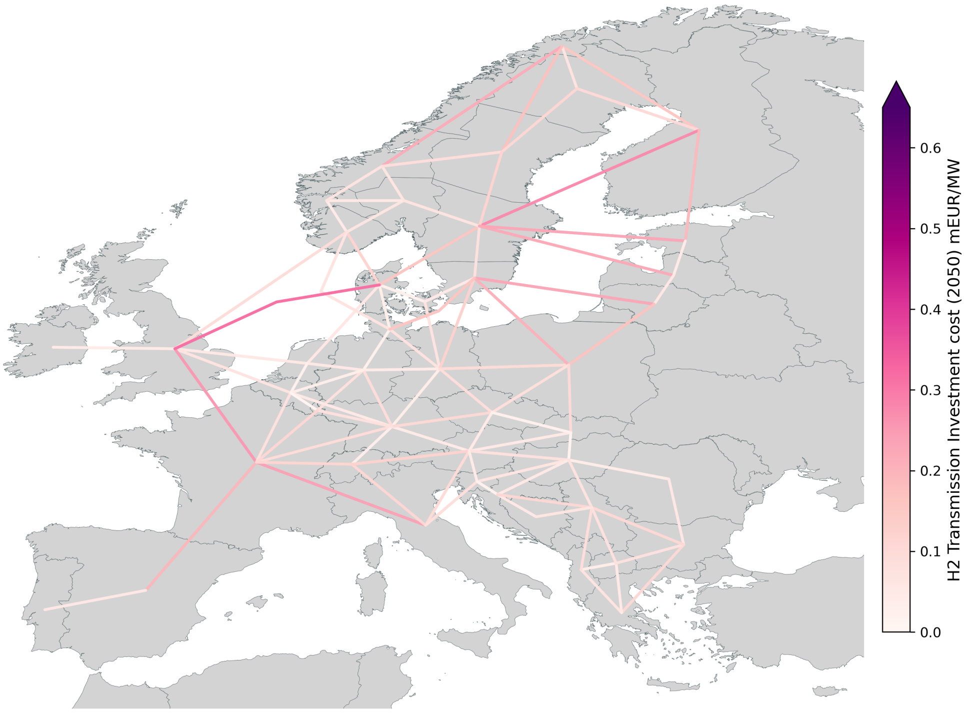

(b) Year: 2050

Supplementary Fig. 3: Hydrogen grid expansion costs. **a**, 2030. **b** 2050.

Supplementary Method 6

Alternative means for hydrogen transportation

The only means of transporting hydrogen through European countries, which we provide in this commentary, is in a gas form and through utilizing existing natural gas infrastructure or newly built pipelines. Other transportation means through shipping or trucks in the form of ammonia, methanol, liquid hydrogen, liquid organic hydrogen carriers (LOHC), and methane may be possible in the future. We compare long-distance hydrogen transportation on the basis of transportation costs.

Carbon-containing carriers (such as methanol or methane) require a sustainable carbon source (biogenic or directly from the air) to be termed renewable, and the cost advantages are insufficient to compensate for this disadvantage [10]. The cost of a hydrogen pipeline for an average distance of 1000 km varies depending on whether it is new or repurposed, ranging from 0.15 - 0.35 €/kg to 0.075 - 0.11 €/kg [11]. Many recent studies debate whether transportation through ammonia or LOHC is more competitive. Di Lullo et al. [12] demonstrate that 1000 km new hydrogen pipelines exhibit a lower transportation cost of approximately 0.41 €/kg compared to LOCH 2.45€/kg and ammonia 2.73 €/kg. Furthermore, Di Lullo et al. [12] show that transportation of hydrogen via trucks in gaseous or liquid form is more expensive than 6 €/kg and suggest that when marine transport is required due to the need of gaseous hydrogen for liquefaction, ammonia or LOCH can be attractive. By 2050, IRENA [10] forecasts a drop in hydrogen transport costs via ammonia and LOCH to 1.3 - 0.75 €/kg and 1.6 - 1.25 €/kg respectively, down from higher costs in 2030, for a reference distance of 10000 km. According to IRENA [10], trucks can be recruited for small volumes and short distances at a cost ranging from 0.55 - 2.11 €/kg, which are lower figures than Di Lullo et al. [12]. However, IRENA emphasizes that due to the significant volume of cross-border trade, trucks are not an appealing option. Additionally, the global trade of ammonia and direct use lowers the need to convert it to hydrogen. LCOH and liquid hydrogen lack this advantage. Similar trends and transportation cost estimates are presented in ref. [11]. Another study by Moritz et al. [13] investigates optimal pathways for transporting hydrogen to Germany, concluding that transporting liquid commodities over a distance of 200–300 km is more competitive than the corresponding pipeline alternative. Overall, liquid fuel transportation cost estimates reflect significant logistics and infrastructure improvements.

Furthermore, transportation cost uncertainty should account for geospatial variations, which can significantly impact production costs [14], or other characteristics such as ports and regulation readiness to accommodate international hydrogen trade [15]. Transporting hydrogen through pipelines is a mature technology with more than 4600km established in the United States and Europe and presents synergies with underground hydrogen storage in salt caverns [10]. Yet the technology may exhibit technical challenges for utilizing existing gas infrastructure [16, 17]. Our review indicates that within Europe, pipeline transportation of hydrogen is more cost-effective than shipping, especially for distances under 4000 km [10]. This suggests that leveraging the potential lower hydrogen production costs in North Africa [13, 18], coupled with the cost advantages of repurposing existing natural gas pipelines, position North

African gas hydrogen imports as a competitive import option for Europe. A recent study [19] assessing site-specific gas hydrogen and derivatives import costs further supports the above conclusion, provided that the European hydrogen network will be available in time for transport. The study further emphasizes that countries like Brazil, Colombia, and Australia might offer competitive import conditions for liquid hydrogen, ammonia, and methanol. Repurposing or building new fuel infrastructure in ports would, however, need to be taken into account to assess the competitiveness of that solution, which was outside the scope of this study.

Supplementary Method 7

Salt cavern simulation

Details about the simulation model for salt caverns can be found in the main article's methods section. Supplementary Table 2 presents the simulation of a 1 TWh capacity cavern. For a 24-hour operation, a maximum pressure drop of 10 bar is permitted. We calculate that the maximum hourly discharge is roughly 162 ton/hr. The cavern operation should maintain a pressure range of 180 to 105 bar and a constant temperature of 39 °C. We estimate that it takes about 185 hours, assuming steady operation, to fully discharge 1 TWh of hydrogen.

Supplementary Table: 2: Salt cavern underground storage simulation of 1 TWh. The pressure inside the cavern should be higher than 110 barg and lower than 180 barg, aiming at approximately 10 barg difference every 24 hours of continues operation (injection or withdrawal cycle) maintaining the structural integrity of the cavern.

Volume [Gwh]	Temp [°C]	p [barg]	H2 density [kg/m3]	H2 mass [ton]	Difference [ton]	Difference every 10 bar	Discharge kg/s	
1071.33	39.00	71.00	5.30	32172.08	2168.77	4324.80	45.12	
1143.55	39.00	76.00	5.65	34340.86	2156.02	-		
1215.35	39.00	81.00	6.01	36496.88	2143.87	4275.60	Discharge ton/hr	
1286.74	39.00	86.00	6.36	38640.75	2131.73	-	162.43	
1357.72	39.00	91.00	6.71	40772.48	2119.58	4227.02		
1428.31	39.00	96.00	7.06	42892.06	2107.43	-	Discharge MWh/hour	
1498.48	39.00	101.00	7.41	44999.50	2095.29	4178.43	5413.92	
1568.26	39.00	106.00	7.75	47094.78	2083.14	-	-	Ton discharge
1637.62	39.00	111.00	8.10	49177.92	2071.60	4131.06	25.43	
1706.61	39.00	116.00	8.44	51249.53	2059.46	-	-	
1775.19	39.00	121.00	8.78	53308.98	2047.92	4083.69	25.14	
1843.38	39.00	126.00	9.11	55356.90	2035.77	-		
1911.18	39.00	131.00	9.45	57392.67	2024.84	4038.74	24.86	
1978.60	39.00	136.00	9.78	59417.51	2013.91	-		
2045.67	39.00	141.00	10.12	61431.41	1998.12	3990.16	24.56	30030.03
2112.20	39.00	146.00	10.44	63429.53	1992.04	-		
2178.54	39.00	151.00	10.77	65421.57	1979.90	3947.64	24.30	
2244.47	39.00	156.00	11.10	67401.46	1967.75	-		
2309.99	39.00	161.00	11.42	69369.21	1955.60	3899.06	24.00	
2375.12	39.00	166.00	11.74	71324.81	1943.46	-		
2439.83	39.00	171.00	12.06	73268.27	1931.31	3856.54	23.74	
2504.15	39.00	176.00	12.38	75199.58	1925.24	-	-	
2568.26	39.00	181.00	12.70	77124.81	1913.09	3814.03	-	
2631.96	39.00	186.00	13.01	79037.90	1900.94	-		
2695.26	39.00	191.00	13.33	80938.84	1888.80	3771.52		
2758.16	39.00	196.00	13.64	82827.64	1882.72			
2820.86	39.00	201.00	13.95	84710.36	3358.53			
2932.69	39.00	210.00	14.50	88068.90	3686.49			
	39.00	220.00	15.11	91755.39				
Hours that you can supply Hydrogen			Cavern volume [m3]					
hr			6073298.15					
184.88								
days								
7.70								
kWh	Mwh	GWh	TWh					
1000000000.00	1000000.00	1000.00	1.00					

Supplementary Method 8

Network expansion: Electricity

The electricity network is modeled with a net transfer capacity similar to ref. [20]. Transmission losses are considered, and distribution losses for generation and storage technologies follow the assumptions of ref. [21]. The investment expenditures are extracted from ref. [22] (see table 3.4 in the report) and are compared with the most recent Danish Energy Agency (DEA) estimates [3]. We distinguish between overground and submarine cables, similar to ref. [2]. High-voltage alternating current (HVAC) is assumed to interconnect synchronous regions through land and high-voltage direct current (HVDC) for the rest. Lastly, the transmission lines are assumed to have a lifetime of 40 years. The following Figures 4a and 4b map the electricity network reinforcement costs. Lastly, Supplementary Fig. 5 illustrates the optimized network development. The network expansion is limited to TYNDP projections for 2035 until that year (see section: Scenario choice and description, Main Manuscript). The model then determines optimal capacities but is limited to a total additional capacity of up to 10 GW per cross-border line connection. Compared to 2020, the results indicate that the electricity network could grow by a factor of four by 2050. Minor variations are noted among the scenarios.

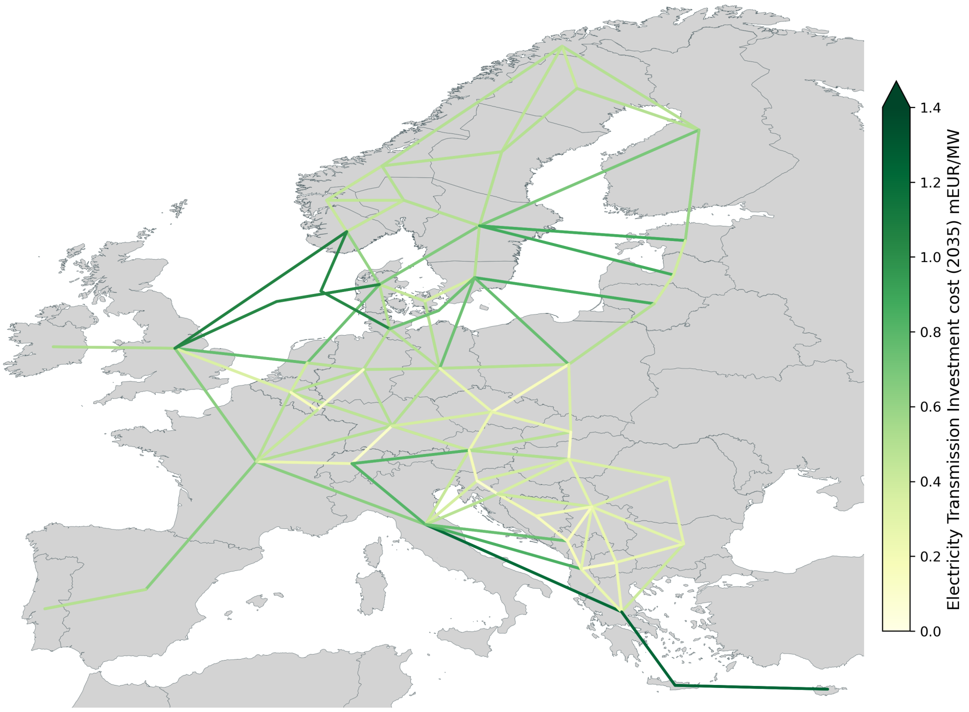

(a) Year: 2035

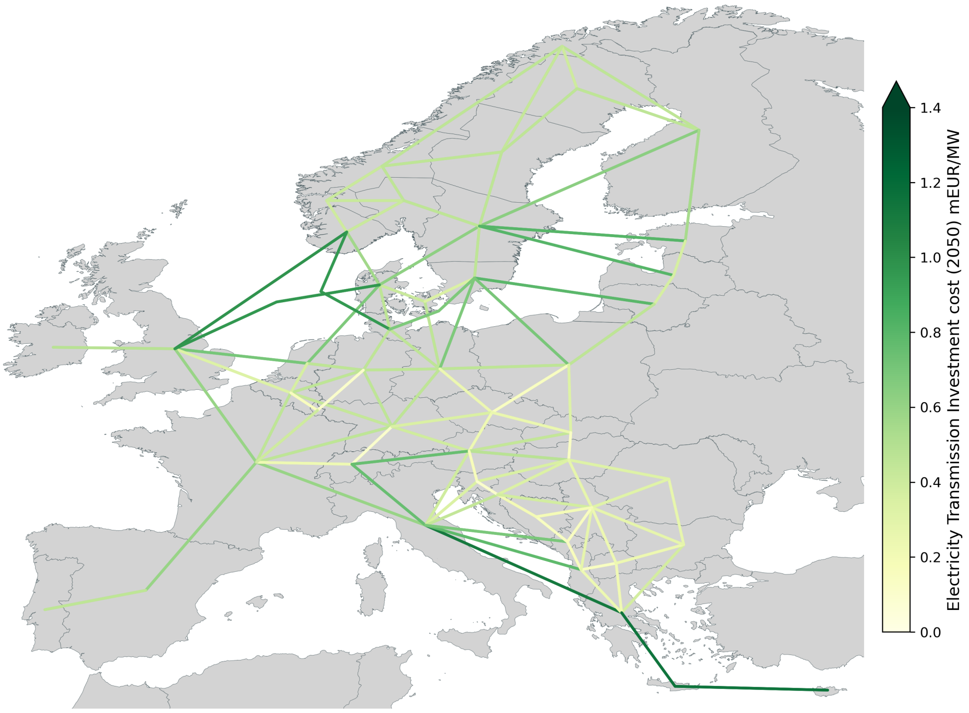

(b) Year: 2050

Supplementary Fig. 4: Electricity grid expansion costs. **a**, 2035. **b**, 2050.

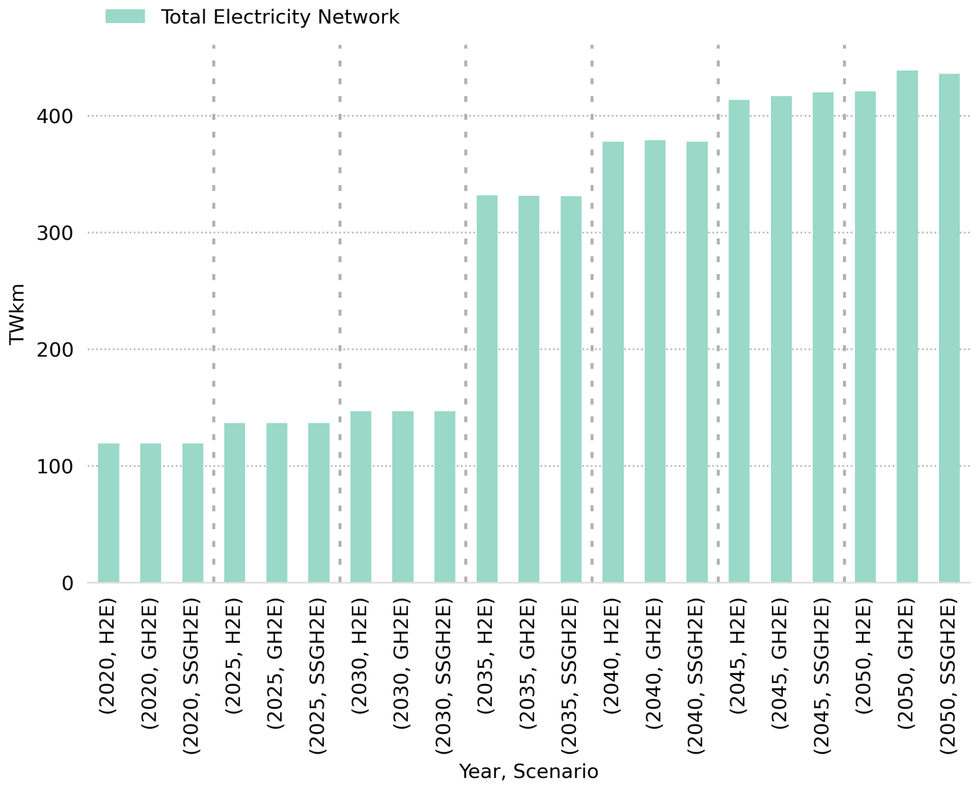

Supplementary Fig. 5: Crossborder electricity network capacity expansion (TWkm) under the three main scenarios Hydrogen Europe (H2E), Green Hydrogen Europe (GH2E), Self-Sufficient Green Hydrogen Europe (SSGH2E).

Supplementary Method 9

Wind and solar modeling

We follow a similar methodology approach for modeling variable renewable generation as described in ref. [2] to update the geographical coverage of variable renewable production in the model. To account for renewable variability within a modeled region, we divide renewable potential investments into sub-areas, so-called resource grades [2]. In addition, we allocate unique features per grade, such as maximum renewable investments reflecting land availability, social acceptance, technical limitations, and costs, or other technical characteristics such as full load hours (FLH). Thus, variable renewable potential installations per resource grade can reach a technical limit. National potentials for Solar PV, onshore and offshore wind (Supplementary Fig 6) are extracted and cross-validated across three sources: atlite [23], TransetBW [24] and ENSPRESO database [25].

The resource grades for offshore wind take into consideration the distance to shore and grid connection (AC or DC). We assume three different types of resource grades. For instance, resource grade type 1 distributes potential investments to near-shore locations, resource grade type 2 reflects investments for offshore wind farms and assumes an AC connection, and resource grade type 3 accounts for DC-connected assets used for the most remote offshore locations possible. Cost estimates are based on the ref. [26]. In principle, resource grade type 3 has more significant capital expenditure costs than resource grade type 1 or 2 but possesses higher capacity factors due to the higher and more stable wind speeds further offshore.

For both solar PV and onshore wind investments, resource grades reflect potential renewable installations and characteristics of renewable generation (i.e. capacity factors and FLH). The costs of capital expenditures are presumed to be the same across all grades. Additionally, we distinguish three categories here. Resource grade type 1 consists of the 10 % of locations with the highest mean wind speed, 40 % of the second-best locations are classified as type 2, and the remaining 50 % are classified as type 3.

This study utilizes the Correlations in renewable energy sources (CorRES) simulation model [27] to update the variable time series and, thus, capacity factors for wind and solar PV technologies. The most recent CorRES dataset (ref. [28], version 3) consists of Pan-European hourly solar PV and wind time series. The updated CorRES model utilizes multiple reanalysis databases. For more information, see ref. [29]. Furthermore, the validated dataset has been recently utilized by the European Network of Transmission System Operators for Electricity (ENTOS-E) in a variety of publications, including ERAA 2021 [30] and TYNDP 2022 [31].

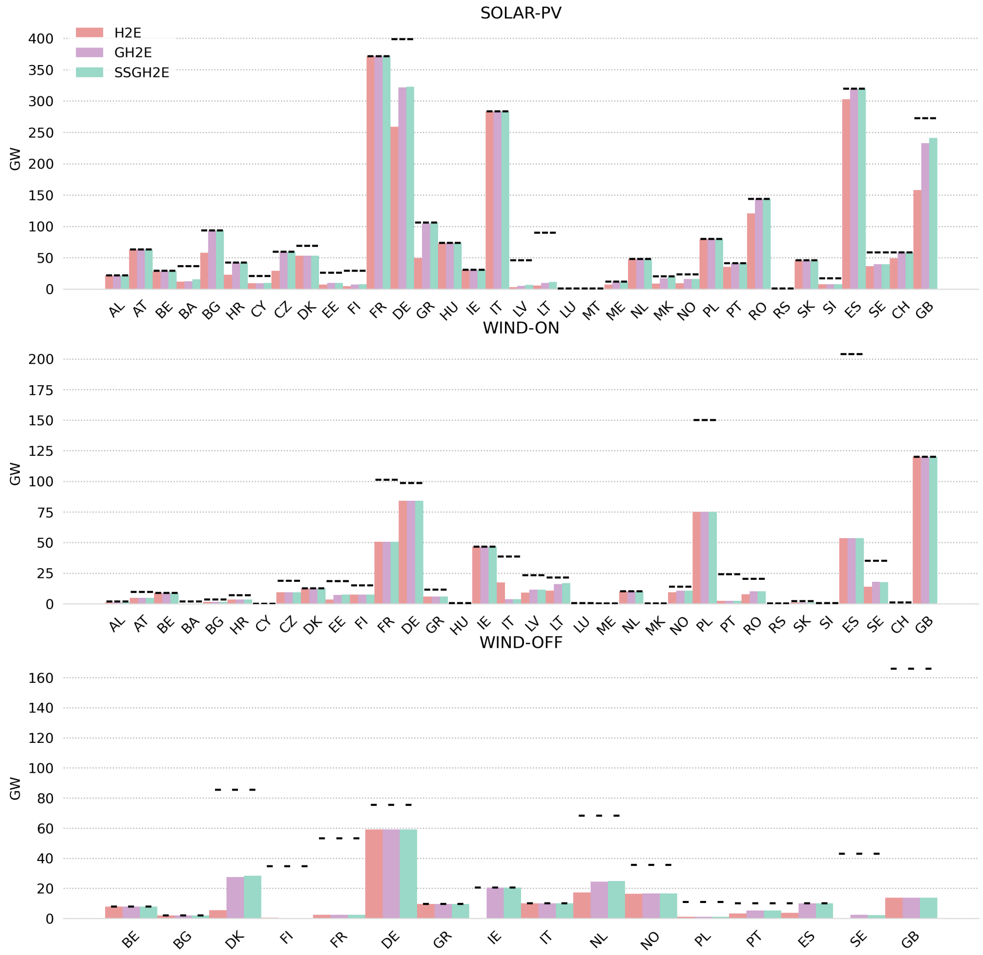

Supplementary Fig. 6: Variable renewable total installed capacity by 2050, three main scenarios Hydrogen Europe (H2E), Green Hydrogen Europe (GH2E), Self-Sufficient Green Hydrogen Europe (SSGH2E), slash symbol indicated the maximum potential installations per country, for solar-PV, wind onshore and wind offshore technologies.

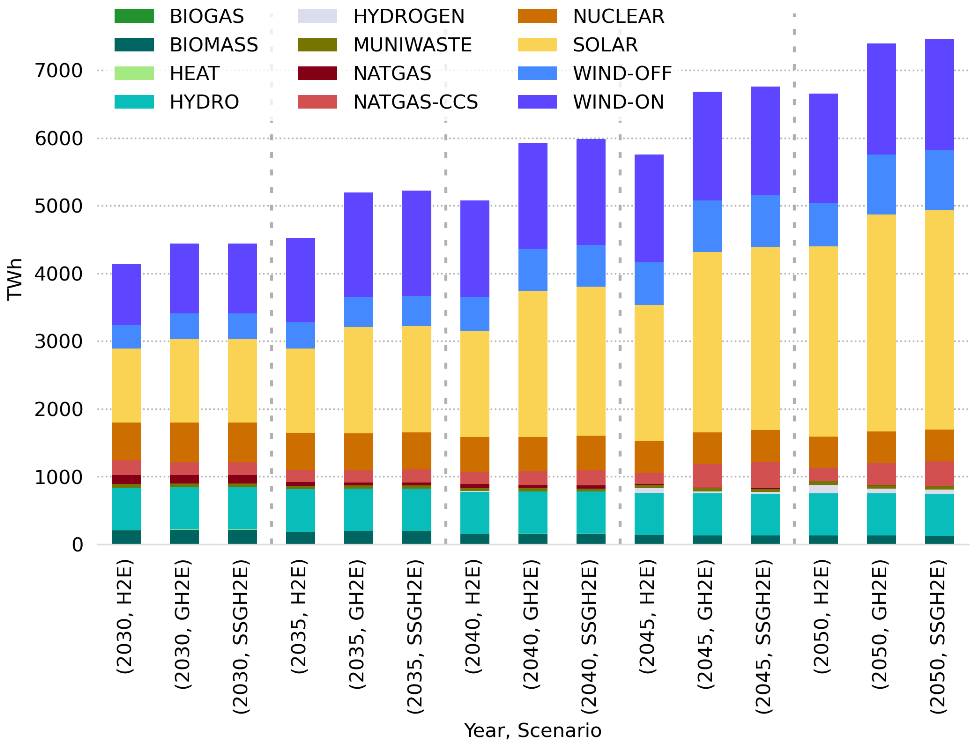

Supplementary Fig. 7: Optimal electricity generation mix pathway for the Hydrogen Europe (H2E), Green Hydrogen Europe (GH2E), Self-Sufficient Green Hydrogen Europe (SSGH2E) scenarios.

Supplementary Method 10

Balmorel European hydrogen demand

Hydrogen is currently primarily used for non-energy-related purposes in industry, but its use as an energy carrier in final demand sectors and power generation is expected to increase in the future. By 2040, global and EU demand for hydrogen and its derivatives in end-use sectors is projected to surpass current non-energy demand. Yet, it's important to consider non-energy uses of hydrogen in understanding demand, as it is currently used in refineries and other industries. Approximately 87 Mt are used globally at this time (2020), while 8 Mt is used in the EU [32].

From the standpoint of modeling European hydrogen infrastructure, the geographical and temporal distribution of exogenous hydrogen demand in the model's region is essential and serves as the primary motivator of investments in hydrogen and network expansion. Yet, forecasting the future European hydrogen demand can be challenging

due to the competition with direct electrification technologies, which remains uncertain. We observe a final demand for hydrogen to be approximately 337 TWh or 10 Mt by 2030 and 1,791 TWh or 53 Mt by 2050. Supplementary Figures 9a - 9e depict the final hydrogen demand evolution across the modeling regions. Our exogenous assumptions for hydrogen penetration over different sectors are based on the detailed hydrogen backbone report [33]. The report provides country-level hydrogen demand and its penetration into different uses such as ammonia synthesis, liquid fuels and high-value chemicals, high-temperature industrial process heat, and iron ore reduction with direct use of hydrogen. These projections are later down-scaled to model regions based on geographical information mapping of European industrial (e.g., iron and steel, cement, chemical industry, refineries) [34] (see Supplementary Fig. 8a) and information for long-haul truck activities [35, 36] (see Supplementary Fig. 8b). The model endogenously optimized the direct hydrogen demand for peak power production. Supplementary Table 3 illustrates the results per scenario. The final exogenous hydrogen demand allocation can be found in Supplementary Table 4.

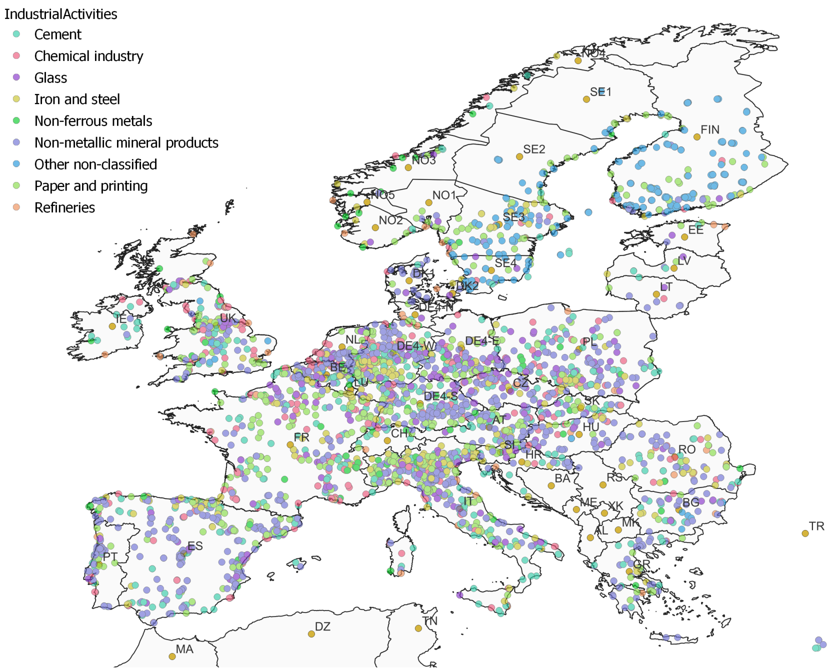

(a)

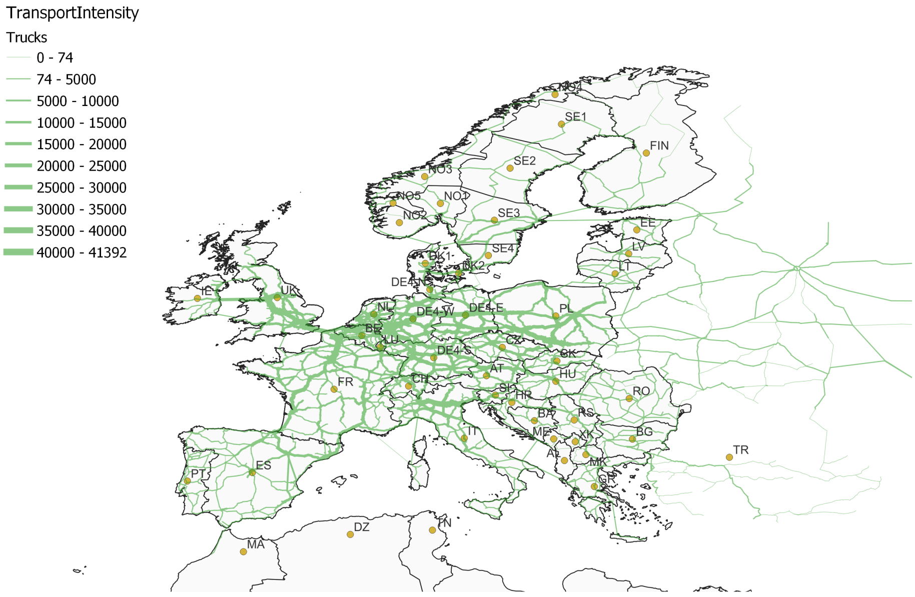

(b)

Supplementary Fig. 8: **a**, Industrial activities 2021 [34]. **b**,Truck activity 2021 [35].

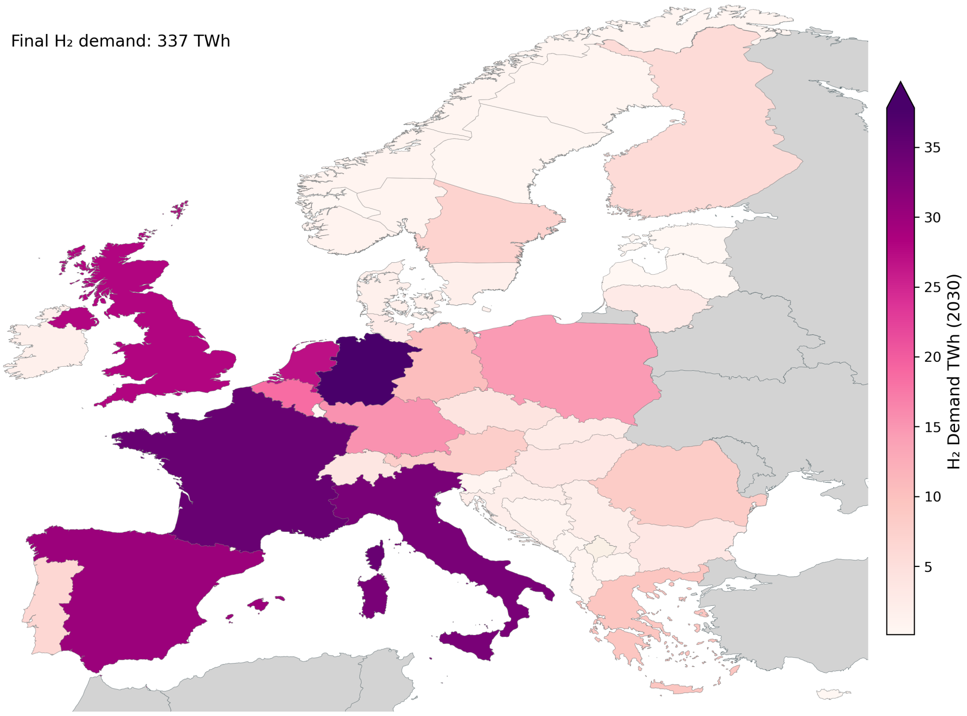

(a)

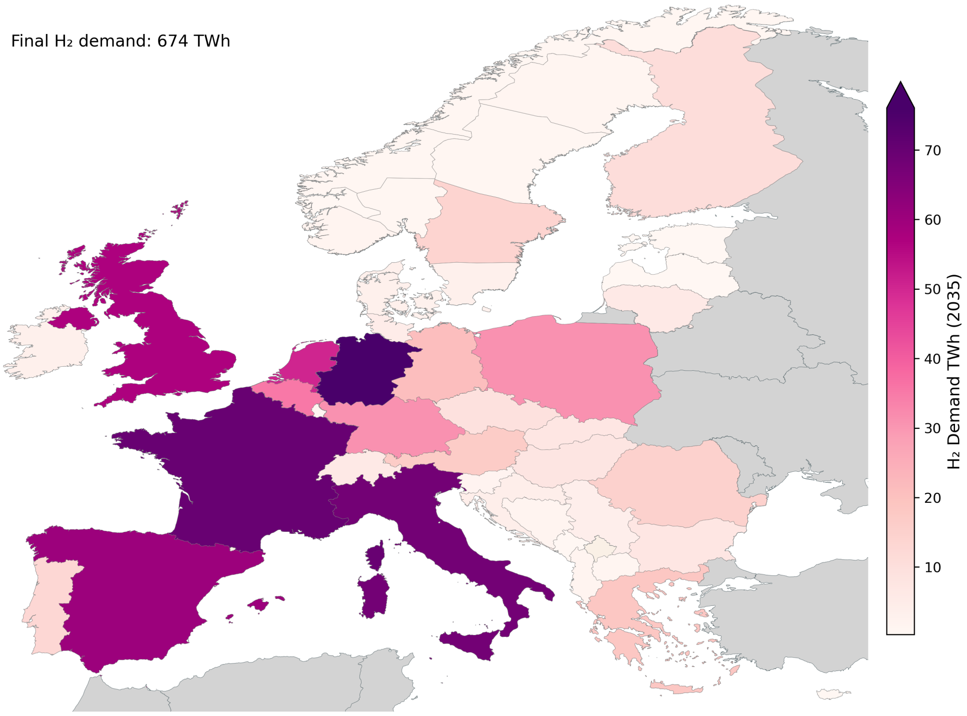

(b)

Supplementary Fig. 9: Hydrogen Europe (H2E) scenario, Hydrogen, exogenous and endogenous final demand: **a**, 2030. **b**, 2035.

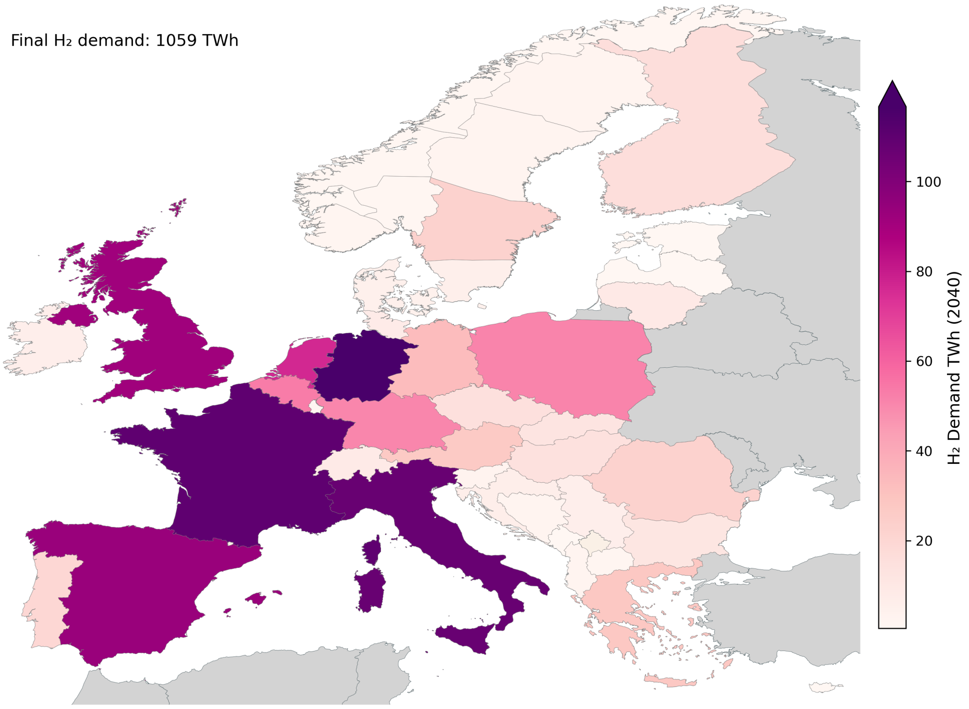

(c)

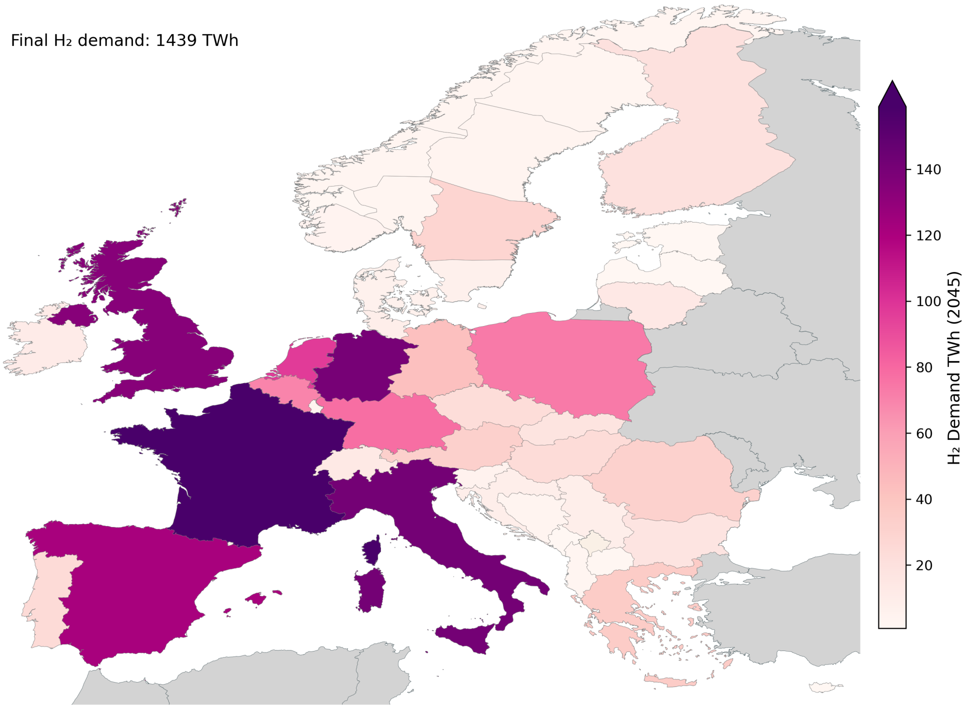

(d)

Supplementary Fig. 9: Hydrogen Europe (H2E) scenario, Hydrogen, exogenous and endogenous final demand: **c**, 2040. **d**, 2045.

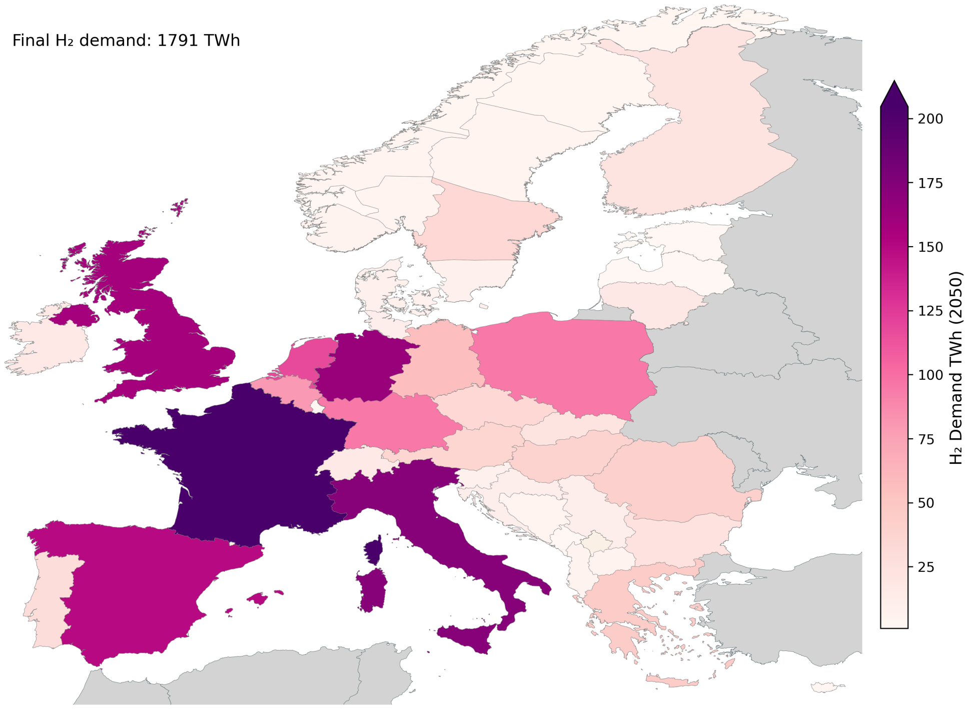

(e)

Supplementary Fig. 9: Hydrogen Europe (H2E) scenario, Hydrogen, exogenous and endogenous final demand: **e**, 2050.

Supplementary Table: 3: Hydrogen to Power, endogenous results for the Hydrogen Europe (H2E), Green Hydrogen Europe (GH2E), Self-Sufficient Green Hydrogen Europe (SSGH2E) scenarios. Years 2030, 2035 are not presented due to zero deployment.

Hydrogen to Power (TWh)	2040	2045	2050
GH2E	-	36.53	102.21
H2E	5.71	103.91	195.07
SSGH2E	-	29.86	90.44

Supplementary Table: 4: Exogenous hydrogen demands allocation to Balmorel regions.

Demand (TWh)	Direct hydrogen for transport					Ammonia and HVC fuels					Steel and Industrial heat				
Balmorel Regions	2030	2035	2040	2045	2050	2030	2035	2040	2045	2050	2030	2035	2040	2045	2050
BE	0.30	1.00	2.10	3.30	4.20	10.53	21.32	32.11	38.08	44.04	7.33	12.58	17.83	18.55	19.27
DK1	0.16	0.50	1.15	1.85	2.35	1.10	2.11	3.11	3.72	4.34	0.33	0.65	0.97	1.14	1.30
DK2	0.16	0.50	1.15	1.85	2.35	1.10	2.11	3.11	3.72	4.34	0.05	0.10	0.16	0.18	0.21
EE	0.10	0.20	0.40	0.60	0.70	0.00	0.00	0.00	0.00	0.00	0.03	0.07	0.11	0.14	0.16
FIN	0.51	1.30	2.90	4.50	5.80	3.06	5.85	8.64	10.34	12.04	2.17	3.37	4.57	4.72	4.87
FR	3.31	9.80	21.30	33.30	41.50	18.81	37.34	55.86	66.45	77.03	12.11	21.67	31.23	35.24	39.25
DE4-E	0.75	1.87	4.11	6.64	8.61	4.06	8.41	12.76	15.50	18.24	5.39	10.51	15.63	17.82	20.01
DE4-N	0.13	0.32	0.70	1.13	1.46	1.48	3.06	4.64	5.64	6.63	0.59	1.16	1.72	1.98	2.23
DE4-S	1.20	2.99	6.57	10.62	13.77	5.35	11.08	16.81	20.43	24.05	8.52	16.61	24.69	28.17	31.65
DE4-W	1.54	3.83	8.42	13.61	17.65	15.68	32.48	49.28	59.89	70.49	19.91	38.46	57.01	64.41	71.80
UK	2.46	7.20	16.30	27.30	36.70	19.21	38.50	57.79	68.63	79.47	5.61	10.45	15.29	18.35	21.41
NL	1.57	2.10	4.70	8.00	10.80	18.05	36.90	55.74	65.99	76.24	6.98	10.66	14.34	20.26	26.18
LV	0.10	0.30	0.70	1.20	1.50	0.00	0.00	0.00	0.00	0.00	0.04	0.08	0.12	0.14	0.15
LT	0.20	0.60	1.30	1.90	2.20	2.32	4.89	7.45	11.26	15.06	0.21	0.40	0.58	0.66	0.74
NO1	0.22	0.44	0.88	1.45	1.76	0.26	0.29	0.33	0.75	1.18	0.26	0.29	0.33	0.75	1.18
NO2	0.29	0.58	1.15	1.90	2.30	0.39	0.44	0.49	1.13	1.77	0.39	0.44	0.49	1.13	1.77
NO3	0.16	0.33	0.66	1.09	1.32	0.30	0.34	0.38	0.88	1.38	0.30	0.34	0.38	0.88	1.38
NO4	0.26	0.52	1.04	1.71	2.08	0.19	0.22	0.25	0.56	0.88	0.19	0.22	0.25	0.56	0.88
NO5	0.07	0.13	0.27	0.44	0.54	0.06	0.07	0.08	0.19	0.29	0.06	0.07	0.08	0.19	0.29
PL	2.50	7.20	15.50	23.00	26.60	6.99	14.48	21.97	32.19	42.41	4.96	9.10	13.23	15.95	18.66
SE1	0.21	0.60	1.29	2.03	2.57	0.00	0.00	0.00	0.00	0.00	0.43	0.70	0.97	0.98	0.98
SE2	0.37	1.06	2.30	3.61	4.57	0.00	0.00	0.00	0.00	0.00	0.01	0.01	0.01	0.02	0.02
SE3	1.02	2.95	6.38	10.02	12.70	4.14	7.93	11.71	14.01	16.32	1.88	3.07	4.25	4.30	4.34
SE4	0.31	0.90	1.94	3.04	3.86	0.96	1.83	2.70	3.23	3.77	0.44	0.72	1.01	1.02	1.03
IT	2.20	6.70	13.80	20.10	23.40	20.57	39.71	58.85	70.33	81.80	9.77	20.57	31.37	35.76	40.14
AT	0.30	0.90	2.10	3.80	5.60	2.62	5.28	7.94	9.42	10.89	4.89	10.67	16.45	17.29	18.12
CZ	0.73	1.60	3.40	4.90	5.70	2.14	4.27	6.40	8.60	10.80	1.29	3.33	5.37	6.66	7.95
CH	0.51	1.00	2.00	4.00	5.00	3.08	4.77	6.46	8.15	9.83	0.00	0.00	0.00	0.00	0.00
ES	1.90	5.50	11.90	18.70	23.70	18.92	37.33	55.74	67.14	78.53	8.73	16.37	24.00	26.53	29.05
PT	0.30	0.90	2.00	3.20	4.10	5.26	10.06	14.86	17.79	20.71	0.85	1.66	2.46	2.84	3.22
SK	0.40	1.10	2.40	3.50	4.10	1.52	3.14	4.75	6.93	9.11	0.77	2.96	5.14	6.74	8.34
HU	0.40	1.30	2.70	4.50	6.10	2.02	4.15	6.28	9.04	11.80	0.91	2.31	3.70	4.57	5.44
SI	0.40	1.10	2.40	3.50	4.10	0.00	0.00	0.00	0.00	0.00	0.34	0.67	0.99	1.15	1.30
HR	0.10	0.30	0.70	1.00	1.30	1.64	3.31	4.98	6.88	8.78	0.25	0.48	0.71	0.82	0.93
RO	0.40	1.10	2.30	3.40	4.20	2.91	6.46	10.01	16.71	23.41	4.95	7.29	9.63	10.32	11.01
BG	0.30	0.90	1.80	2.60	2.90	2.39	4.94	7.49	10.96	14.42	0.69	1.32	1.95	2.24	2.52
GR	0.30	1.00	2.00	3.00	3.80	8.82	16.93	25.04	30.31	35.58	0.18	0.35	0.51	0.59	0.66
IE	0.20	0.80	2.30	6.00	11.20	0.87	1.66	2.45	2.94	3.42	0.49	0.97	1.44	1.68	1.91
LU	0.10	0.20	0.50	1.10	1.90	0.00	0.00	0.00	0.00	0.00	0.26	0.51	0.75	0.86	0.96
AL	0.00	0.00	0.00	0.00	0.00	0.45	0.87	1.29	1.56	1.83	0.47	0.91	1.34	1.62	1.90
ME	0.00	0.00	0.00	0.00	0.00	0.21	0.41	0.61	0.74	0.86	0.22	0.43	0.63	0.76	0.90
MK	0.00	0.00	0.00	0.00	0.00	0.51	0.97	1.44	1.74	2.04	0.53	1.01	1.49	1.80	2.11
BA	0.00	0.00	0.00	0.00	0.00	0.60	1.21	1.82	2.52	3.21	0.78	1.56	2.34	3.12	3.89
RS	0.00	0.00	0.00	0.00	0.00	1.14	2.35	3.57	5.22	6.87	1.80	3.61	5.42	7.35	9.27
MT	0.00	0.00	0.00	0.00	0.00	0.00	0.00	0.00	0.00	0.00	0.00	0.00	0.00	0.00	0.00
CY	0.10	0.30	0.70	1.00	1.30	0.00	0.00	0.00	0.00	0.00	0.00	0.00	0.00	0.00	0.00

Supplementary Method 11

European hydrogen demand scenarios

In our analysis, we compare European hydrogen demand projections from various sources, including studies by Tarvydas et al. (2022) [32] as well as scenarios documented in the European Hydrogen Observatory database (2023) [37]. The projections show minor discrepancies for the year 2030 (Supplementary Fig. 10a) but diverge significantly for 2050 (Supplementary Fig. 10b). Notably, the RePowerEU initiative emerges as a particularly ambitious plan by 2030, with a strong focus on hydrogen integration within the transport sector. A comparative analysis of the decomposition of hydrogen demand across different sectors also reveals variations in the projections for 2050. Our findings indicate that the projected hydrogen-to-power deployment by 2050, ranging from 2.7 to 6 Mt (Supplementary Table 3) is consistent with current trends and aligns with the European Commission's Fit-for-55 package. By 2050, most studies anticipate a total hydrogen demand between 20 to 64 Mt, averaging around 41 Mt, indicating a significant potential increase in hydrogen utilization.

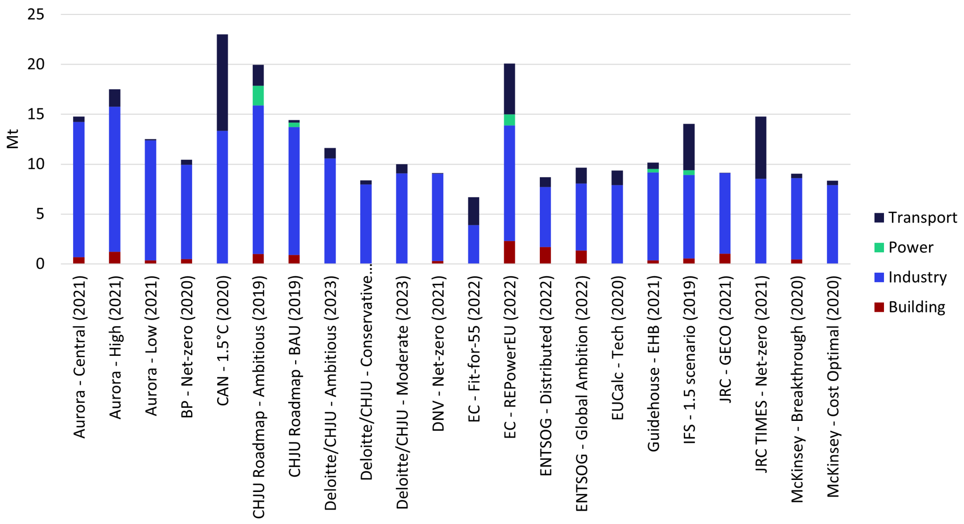

(a)

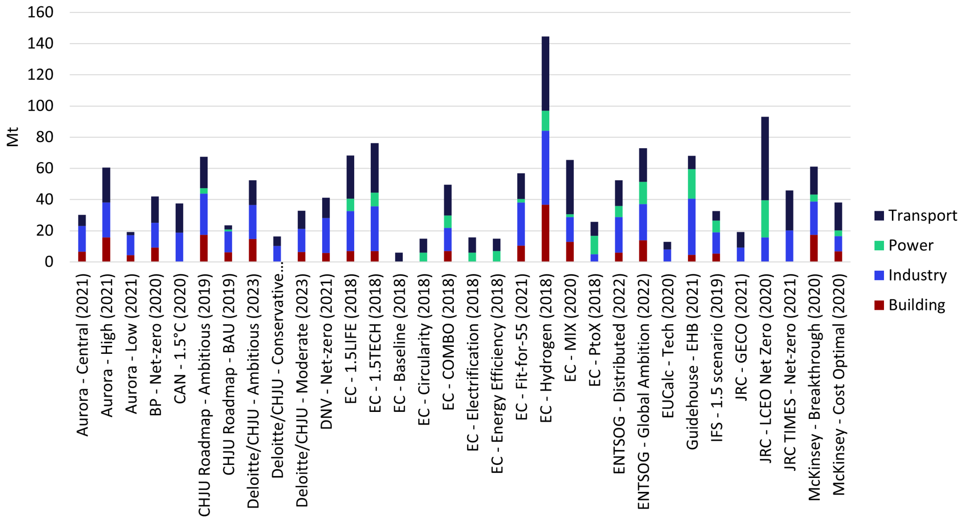

(b)

Supplementary Fig. 10: Scenarios for future European hydrogen demand development adopted data from ref. [37]: **a**, 2030. **b**, 2050. For further information about the sectoral scope and key assumptions, see ref. [37], 1 Mt = 33.33 TWh.

Supplementary Method 12

Importing hydrogen from third nations via pipelines

The most recent European Hydrogen Backbone (EHB) report [38] provides data on potential hydrogen import volumes to the European system (Supplementary Table 5). These targets are used to estimate the future costs of producing and transporting hydrogen to the system boundaries of the Balmorel model. To accomplish this, an optimization model of planning and operations is developed in order to provide information on the optimally installed capacities of renewable assets, water-alkaline electrolysis, and hydrogen pipeline capacities. We do not offer hydrogen storage investment opportunities. We presume the produced hydrogen can be injected directly into the pipeline network. In addition, for consistency, we use the same data on technology cost development and a 4% discount rate as in the main Balmorel model. On the basis of the optimal decisions for long-term planning, we estimate the cost of producing and transporting hydrogen. The following Supplementary Tables 7, 8 and 9 provide an extensive estimate of the prospective cost of producing and transporting hydrogen to the system entry points Tunisia & Algeria - Italy, Morocco - Spain, Ukraine - Slovakia. The costs are summed up in Supplementary Table 6 and are used as fuel prices in the main model. The optimal hydrogen pipeline capacities from third nations to the European border constrain the hourly imported hydrogen volume at the entry point.

Supplementary Table: 5: Maximum hydrogen supply base on ref. [38].

H2 (TWh/a)	2030	2040	2050
Tunisia and Algeria	70	150	375
Morocco	-	46	115
Ukraine	12	50	100

Supplementary Table: 6: Cost of producing and transporting hydrogen to Balmorel system boundaries, assuming a 4% discount rate. A linear interpolation is used for the years 2035 and 2045.

Hydrogen import (€/kg)	2030	2040	2050
Tunisia and Algeria	1.30	0.99	0.76
Morocco	-	1.01	0.79
Ukraine	1.80	1.53	1.37

Supplementary Table: 7: Cost of producing and transporting hydrogen, importing from Tunisia and Algeria.

PV	Units	2030	2040	2050
Capacity installed	MW	57089.00	67925.43	188340.39
FLH	h/y	1916.20	1835.67	1916.19
Production EL	GWh/y	109394.00	124689.00	360896.00
CAPEX	€/KW	380.00	320.00	290.00
discount rate	%	0.04	0.04	0.04
interest	%	0.04	0.04	0.04
debt share	%	0.00	0.00	0.00
Lifetime	y	30.00	30.00	30.00
Annuity	-	0.06	0.06	0.06
Annual capital cost	Mio. €/y	1254.56	1257.00	3158.61
Spec. Opex	€/y as % of capex	2.00	2.00	2.00
Opex Cost PV	Mio. €/y	25.09	25.14	63.17
Total Annual Cost PV	Mio. €/y	1279.65	1282.14	3221.78
LCOE	€/KWh	0.01	0.01	0.01

LCOE	€/MWh	11.70	10.28	8.93
Wind offshore	Units	2030	2040	2050
Capacity installed	MW	0.00	0.00	0.00
FLH	h/y	0.00	0.00	0.00
Production EL	GWh/y	0.00	0.00	0.00
CAPEX	€/KW	1800.00	1680.00	1640.00
discount rate	%	0.04	0.04	0.04
interest	%	0.04	0.04	0.04
debt share	%	0.00	0.00	0.00
Lifetime	y	30.00	30.00	30.00
Annuity	-	0.06	0.06	0.06
Annual capital cost	Mio. €/y	0.00	0.00	0.00
Spec. Opex	€/y as % of capex	2.00	2.00	2.00
Opex Cost Wind	Mio. €/y	0.00	0.00	0.00
Total Annual Cost Wind	Mio. €/y	0.00	0.00	0.00
LCOE	€/KWh	0.00	0.00	0.00
LCOE	€/MWh	0.00	0.00	0.00
Electricity produced	GWh/y	109394.00	124689.00	360896.00
Electricity Consumed	GWh/y	100000.00	114286.00	335821.00
Curtail	GWh/y	9394.00	10403.00	25075.00
Electrolysis	Units	2030	2040	2050
Capacity installed	MWe	35858.63	39463.70	106263.63
Electricity Consumed	GWh/y	100000.00	114286.00	335821.00
FLH	h/y	2904.93	2895.97	2857.46
Efficiency LHV	%	0.67	0.70	0.74
Hydrogen Production	GWh/y	70000.00	80000.00	225000.00
CAPEX	€/KW	800.00	650.00	500.00
discount rate	%	0.04	0.04	0.04
interest	%	0.04	0.04	0.04
debt share	%	0.00	0.00	0.00
Lifetime	y	30.00	30.00	30.00
Annuity	-	0.06	0.06	0.06
Annual capital cost	Mio. €/y	1658.97	1483.42	3072.62
Spec. Opex	€/y as % of capex	2.00	2.00	2.00
Opex Cost Electrolysis	Mio. €/y	3.32	2.97	6.15
Sum Anual Cost Electrolysis	Mio. €/y	1662.28	1486.39	3078.76
Cost of Electrolysis investment	€/KWh	0.02	0.02	0.01
Cost of electricity production	€/KWh	0.01	0.01	0.01
LCOH2	€/KWh	0.04	0.03	0.02
LCOH2	€/MWh	35.44	28.86	22.61
LCOH2	€/GJ	9.85	8.02	6.28
LCOH2	€/ton	1.06	0.87	0.68
Hydrogen transport Pipelines	Units	2030	2040	2050
Distance offshore	KM	120.00	120.00	120.00
Distance onshore	KM	100.00	100.00	100.00
CAPEX offshore	€/MW/KM	170.00	145.00	120.00
CAPEX onshore	€/MW/KM	150.00	135.00	120.00
Cost offshore	€/MW	20400.00	17400.00	14400.00
Cost onshore	€/MW	15000.00	13500.00	12000.00
CAPEX Compressor station	Mio.€/Mwe	6.70	3.40	2.20
Total pipes cost per MW	€/MW	35400.00	30900.00	26400.00
Capacity of pipe	MW	24097.00	27624.59	78741.35
discount rate	%	0.04	0.04	0.04
interest	%	0.04	0.04	0.04
debt share	%	0.00	0.00	0.00
Lifetime	y	50.00	50.00	50.00
Annuity	-	0.05	0.05	0.05
Annual Pipe Cap Cost	Mio. €/y	39.71	39.74	96.77
Opex pipe	€/y as % of capex	1.00	0.90	0.80
Opex Cost of pipes	Mio. €/y	0.40	0.36	0.77
Total Compressor Station Cost	Mio. €	10763.33	6261.57	11548.73
Annual Compressor Station Cost	Mio. €/y	501.04	291.48	537.60
Opex Compressor Station	€/y as % of capex	1.70	1.70	1.70
Opex Cost of Compressor station	Mio. €/y	8.52	4.96	9.14
Sum Annual Cost pipes	Mio. €/y	549.66	336.53	644.28
Transported H2/year	GWh/y	70000.00	80000.00	225000.00
LCOT(transport)	€/KWh	0.008	0.004	0.003
LCOT(transport)	€/MWh	7.85	4.21	2.86
LCOT(transport)	€/GJ	2.18	1.17	0.80
LCOT(transport)	€/ton	0.24	0.13	0.09
Total Costs	Units	2030	2040	2050
LCOH2 production	€/MWh	35.44	28.86	22.61
LCOTH2 Transportation	€/MWh	7.85	4.21	2.86
Total LCOH2	€/MWh	43.30	33.07	25.47
Total LCOH2	€/GJ	12.03	9.19	7.08
Total LCOH2	€/kg	1.30	0.99	0.76

Supplementary Table: 8: Cost of producing and transporting hydrogen, importing from Morroco.

PV	Units	2030	2040	2050
Capacity installed	MW	0.00	38910.00	57533.00
FLH	h/y	0.00	1846.16	1846.12

Production EL	GWh/y	0.00	71834.00	106213.00
CAPEX	€/KW	380.00	320.00	290.00
discount rate	%	0.04	0.04	0.04
interest	%	0.04	0.04	0.04
debt share	%	0.00	0.00	0.00
Lifetime	y	30.00	30.00	30.00
Annuity	-	0.06	0.06	0.06
Annual capital cost	Mio. €/y	0.00	720.05	964.87
Spec. Opex	€/y as % of capex	2.00	2.00	2.00
Opex Cost PV	Mio. €/y	0.00	14.40	19.30
Total Annual Cost PV	Mio. €/y	0.00	734.46	984.17
LCOE	€/KWh	0.00	0.01	0.01
LCOE	€/MWh	0.00	10.22	9.27
Wind offshore	Units	2030	2040	2050
Capacity installed	MW	0.00	0.00	0.00
FLH	h/y	4443.62	4443.62	4443.62
Production EL	GWh/y	0.00	0.00	0.00
CAPEX	€/KW	1800.00	1680.00	1640.00
discount rate	%	0.04	0.04	0.04
interest	%	0.04	0.04	0.04
debtshare	%	0.00	0.00	0.00
Life time	y	30.00	30.00	30.00
Annuity	-	0.06	0.06	0.06
Annual capital cost	Mio. €/y	0.00	0.00	0.00
Spec. Opex	€/y as % of capex	2.00	2.00	2.00
Opex Cost Wind	Mio. €/y	0.00	0.00	0.00
Total Annual Cost Wind	Mio. €/y	0.00	0.00	0.00
LCOE	€/KWh	0.00	0.00	0.00
LCOE	€/MWh	0.00	0.00	0.00
Electricity produced	GWh/y	0.00	71834.00	106213.00
Electricity Consumed	GWh/y	0.00	65714.00	98571.00
Curtail	GWh/y	0.00	6120.00	7642.00
Electrolysis	Units	2030	2040	2050
Capacity installed	MWe	0.00	23307.14	33479.08
Electricity Consumed	GWh/y	0.00	65714.00	98571.00
FLH	h/y	0.00	2819.49	2781.36
Efficiency LHV	%	0.67	0.70	0.74
Hydrogen Production	GWh/y	0.00	46000.00	69000.00
CAPEX	€/KW	800.00	650.00	500.00
discount rate	%	0.04	0.04	0.04
interest	%	0.04	0.04	0.04
debt share	%	0.00	0.00	0.00
Lifetime	y	30.00	30.00	30.00
Annuity	-	0.06	0.06	0.06
Annual capital cost	Mio. €/y	0.00	876.11	968.05
Spec. Opex	€/y as % of capex	2.00	2.00	2.00
Opex Cost Electrolysis	Mio. €/y	0.00	1.75	1.94
Sum Anual Cost Electrolysis	Mio. €/y	0.00	877.86	969.99
Cost of Electrolysis investment	€/KWh	0.00	0.02	0.01
Cost of electricity production	€/KWh	0.00	0.01	0.01
LCOH2	€/KWh	0.000	0.029	0.023
LCOH2	€/MWh	0.00	29.31	23.32
LCOH2	€/GJ	0.00	8.14	6.48
LCOH2	€/ton	0.00	0.88	0.70
Hydrogen transport Pipelines	Units	2030	2040	2050
Distance offshore	KM	170.00	170.00	170.00
Distance onshore	KM	100.00	100.00	100.00
CAPEX offshore	€/MW/KM	170.00	145.00	120.00
CAPEX onshore	€/MW/KM	150.00	135.00	120.00
Cost offshore	€/MW	28900.00	24650.00	20400.00
Cost onshore	€/MW	15000.00	13500.00	12000.00
CAPEX Compressor station	Mio.€/Mwe	6.70	3.40	2.20
Total pipes cost per MW	€/MW	43900.00	38150.00	32400.00
Capacity of pipe	MW	0.00	16315.00	24808.00
discount rate	%	0.04	0.04	0.04
interest	%	0.04	0.04	0.04
debt share	%	0.00	0.00	0.00
Lifetime	y	50.00	50.00	50.00
Annuity	-	0.05	0.05	0.05
Annual Pipe Cap Cost	Mio. €/y	0.00	28.97	37.42
Opex pipe	€/y as % of capex	1.00	0.90	0.80
Opex Cost of pipes	Mio. €/y	0.00	0.26	0.30
Total Compressor Station Cost	Mio. €	0.00	3698.07	3638.51
Annual Compressor Station Cost	Mio. €/y	0.00	172.15	169.37
Opex Compressor Station	€/y as % of capex	1.70	1.70	1.70
Opex Cost of Compressor station	Mio. €/y	0.00	2.93	2.88
Sum Annual Cost pipes	Mio. €/y	0.00	204.31	209.97
Transported H2/year	GWh/y	0.00	46000.00	69000.00
LCOT(transport)	€/KWh	0.00	0.00	0.00
LCOT(transport)	€/MWh	0.00	4.44	3.04
LCOT(transport)	€/GJ	0.00	1.23	0.85
LCOT(transport)	€/ton	0.00	0.13	0.09
Total Costs	Units	2030	2040	2050
LCOH2 production	€/MWh	0.00	29.31	23.32
LCOTH2 Transportation	€/MWh	0.00	4.44	3.04
Total LCOH2	€/MWh	0.00	33.75	26.37
Total LCOH2	€/GJ	0.00	9.37	7.32
Total LCOH2	€/kg	0.00	1.01	0.79

Supplementary Table: 9: Cost of producing and transporting hydrogen, importing from Ukraine.

PV	Units	2030	2040	2050
Capacity installed	MW	5621.19	18178.00	23893.00
FLH	h/y	1281.40	1281.55	1281.46
Production EL	GWh/y	7203.00	23296.00	30618.00
CAPEX	€/KW	380.00	320.00	290.00
discount rate	%	0.04	0.04	0.04
interest	%	0.04	0.04	0.04
debt share	%	0.00	0.00	0.00
Lifetime	y	30.00	30.00	30.00
Annuity	-	0.06	0.06	0.06
Annual capital cost	Mio. €/y	123.53	336.40	400.70
Spec. Opex	€/y as % of capex	2.00	2.00	2.00
Opex Cost PV	Mio. €/y	2.47	6.73	8.01
Total Annual Cost PV	Mio. €/y	126.00	343.12	408.72
LCOE	€/KWh	0.02	0.01	0.01
LCOE	€/MWh	17.49	14.73	13.35
Wind offshore	Units	2030	2040	2050
Capacity installed	MW	6016.71	18013.00	22113.00
FLH	h/y	4443.62	4443.62	4443.62
Production EL	GWh/y	14664.00	43901.00	53895.00
CAPEX	€/KW	1040.00	980.00	960.00
discount rate	%	0.04	0.04	0.04
interest	%	0.04	0.04	0.04
debt share	%	0.00	0.00	0.00
Lifetime	y	30.00	30.00	30.00
Annuity	-	0.06	0.06	0.06
Annual capital cost	Mio. €/y	361.87	1020.86	1227.65
Spec. Opex	€/y as % of capex	2.00	2.00	2.00
Opex Cost Wind	Mio. €/y	7.24	20.42	24.55
Total Annual Cost Wind	Mio. €/y	369.10	1041.28	1252.20
LCOE	€/KWh	0.03	0.02	0.02
LCOE	€/MWh	0.00	0.00	0.00
Electricity produced	GWh/y	21867.00	67197.00	84513.00
Electricity Consumed	GWh/y	17910.00	54286.00	67568.00
Curtail	GWh/y	3957.00	12911.00	16945.00
Electrolysis	Units	2030	2040	2050
Capacity installed	MWe	3269.35	9871.15	12233.47
Electricity Consumed	GWh/y	17910.00	54286.00	67568.00
FLH	h/y	5461.99	5499.43	5515.72
Efficiency LHV	%	0.67	0.70	0.74
Hydrogen Production	GWh/y	12000.00	38000.00	50000.00
CAPEX	€/KW	800.00	650.00	500.00
discount rate	%	0.04	0.04	0.04
interest	%	0.04	0.04	0.04
debt share	%	0.00	0.00	0.00
Lifetime	y	30.00	30.00	30.00
Annuity	-	0.06	0.06	0.06
Annual capital cost	Mio. €/y	151.25	371.05	353.73
Spec. Opex	€/y as % of capex	2.00	2.00	2.00
Opex Cost Electrolysis	Mio. €/y	0.30	0.74	0.71
Sum Anual Cost Electrolysis	Mio. €/y	151.56	371.79	354.44
Cost of Electrolysis investment	€/KWh	0.01	0.01	0.01
Cost of electricity production	€/KWh	0.04	0.04	0.04
LCOH2	€/KWh	0.06	0.05	0.04
LCOH2	€/MWh	55.29	48.23	43.67
LCOH2	€/GJ	15.36	13.40	12.13
LCOH2	€/ton	1.66	1.45	1.31
Hydrogen transport Pipelines	Units	2030	2040	2050
Distance offshore	KM	0.00	0.00	0.00
Distance onshore	KM	560.00	560.00	560.00
CAPEX offshore	€/MW/KM	0.00	0.00	0.00
CAPEX onshore	€/MW/KM	150.00	135.00	120.00
Cost offshore	€/MW	0.00	0.00	0.00
Cost onshore	€/MW	84000.00	75600.00	67200.00
CAPEX Compressor station	Mio.€/Mwe	6.70	3.40	2.20
Total pipes cost per MW	€/MW	84000.00	75600.00	67200.00
Capacity of pipe	MW	2197.00	6909.81	9065.00
discount rate	%	0.04	0.04	0.04
interest	%	0.04	0.04	0.04
debt share	%	0.00	0.00	0.00
Lifetime	y	50.00	50.00	50.00
Annuity	-	0.05	0.05	0.05
Annual Pipe Cap Cost	Mio. €/y	8.59	24.32	28.36
Opex pipe	€/y as % of capex	1.00	0.90	0.80
Opex Cost of pipes	Mio. €/y	0.09	0.22	0.23
Total Compressor Station Cost	Mio. €	981.33	1566.22	1329.53
Annual Compressor Station Cost	Mio. €/y	45.68	72.91	61.89
Opex Compressor Station	€/y as % of capex	1.70	1.70	1.70
Opex Cost of Compressor station	Mio. €/y	0.78	1.24	1.05
Sum Annual Cost pipes	Mio. €/y	55.13	98.68	91.53
Transported H2/year	GWh/y	12000.00	38000.00	50000.00
LCOT(transport)	€/KWh	0.005	0.003	0.002
LCOT(transport)	€/MWh	4.59	2.60	1.83
LCOT(transport)	€/GJ	1.28	0.72	0.51
LCOT(transport)	€/ton	0.14	0.08	0.05
Total Costs	Units	2030	2040	2050

LCOH2 production	€/MWh	55.29	48.23	43.67
LCOTH2 Transportation	€/MWh	4.59	2.60	1.83
Total LCOH2	€/MWh	59.89	50.83	45.50
Total LCOH2	€/GJ	16.64	14.12	12.64
Total LCOH2	€/kg	1.80	1.53	1.37

Supplementary Figures

Additional figures, European hydrogen network, production centers, trading

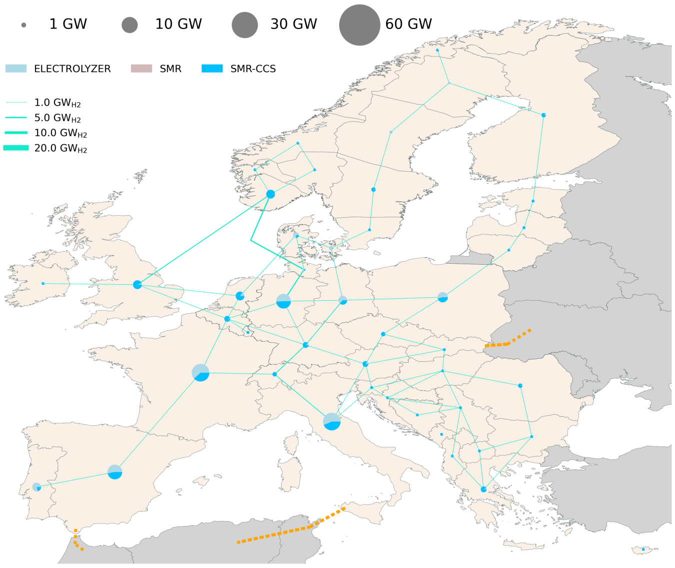

(a)

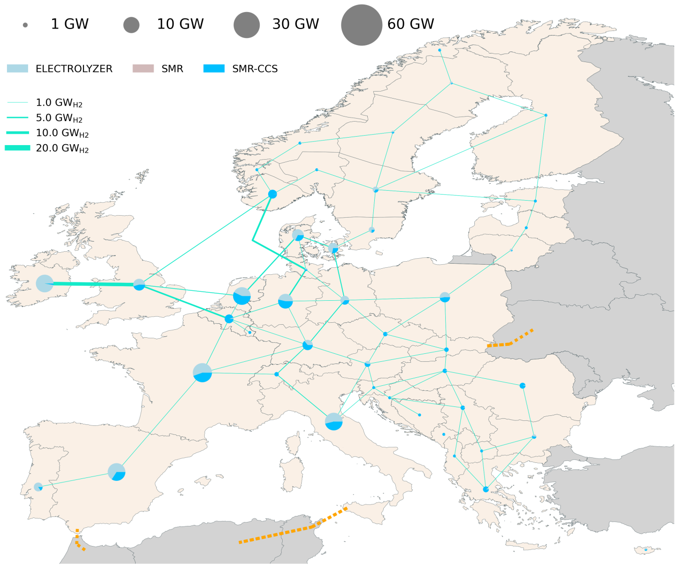

(b)

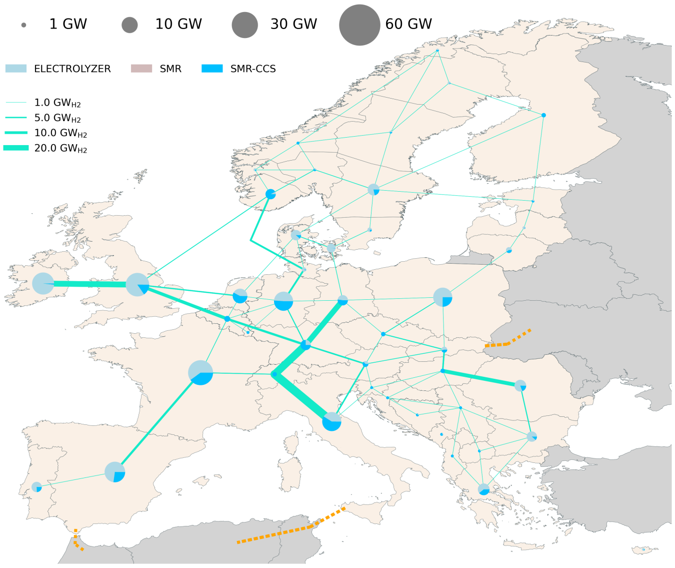

(c)

Supplementary Fig. 11: Hydrogen optimized grid and production centers for Hydrogen Europe (H2E) scenario. **a** 2035, **b** 2040, **c** 2045. The 2030 and 2050 figures are displayed in the main manuscript.

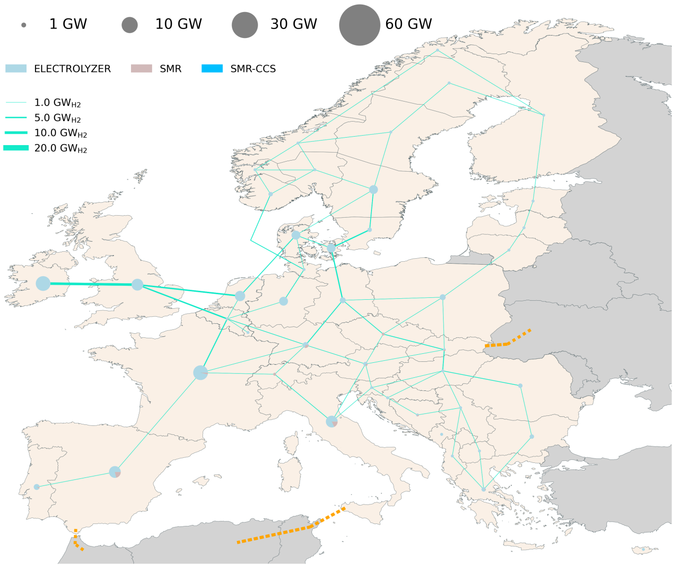

(a)

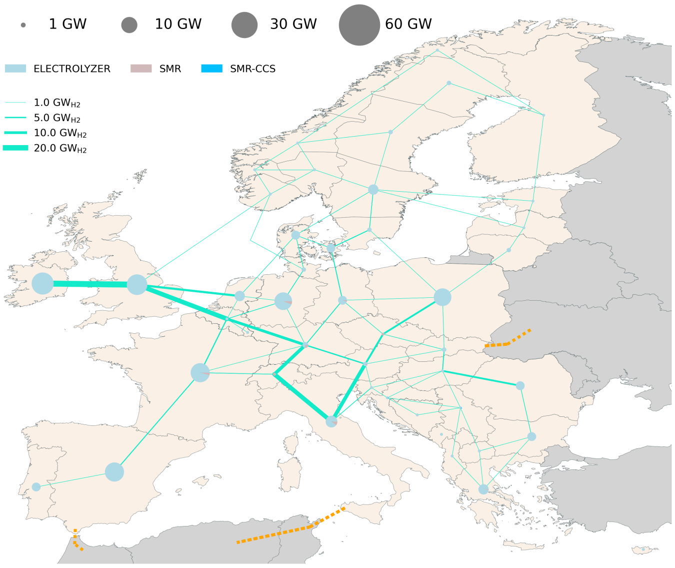

(b)

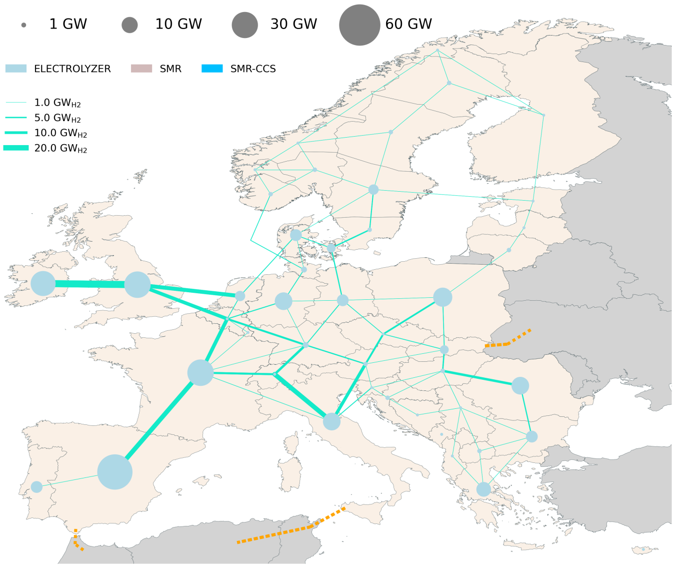

(c)

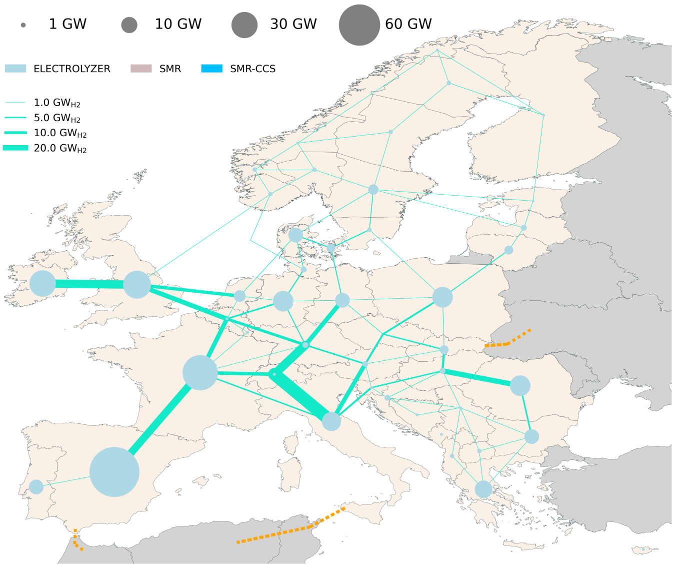

(d)

Supplementary Fig. 12: Hydrogen optimized grid and production centers for Green Hydrogen Europe (GH2E) scenario. **a** 2030, **b** 2035, **c** 2040, **d** 2045. The 2050 figure is displayed in the main manuscript.

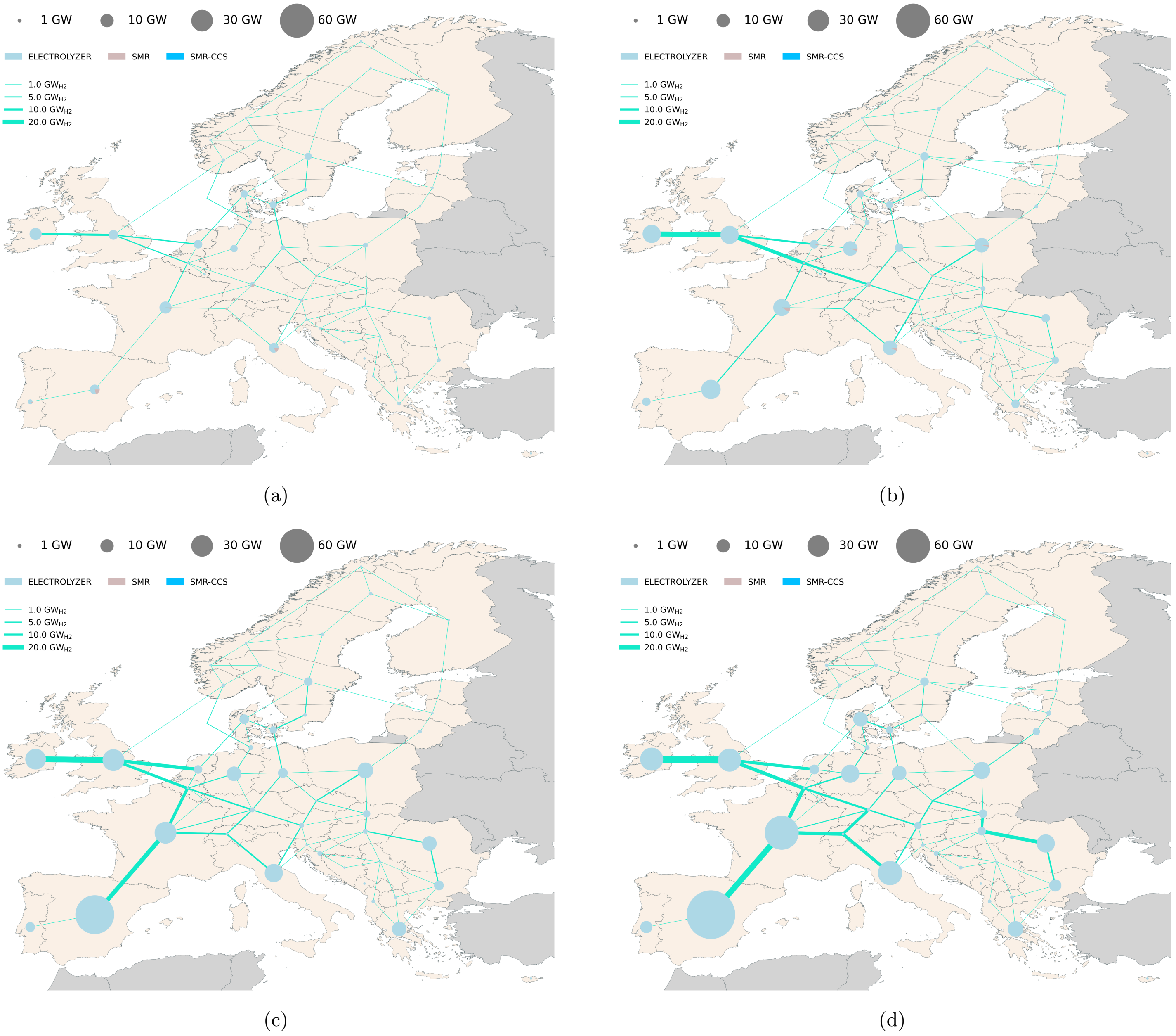

Supplementary Fig. 13: Hydrogen optimized grid and production centers for Self-Sufficient Green Hydrogen Europe (SSGH2E) scenario. **a** 2030, **b** 2035, **c** 2040, **d** 2045. The 2050 figure is displayed in the main manuscript.

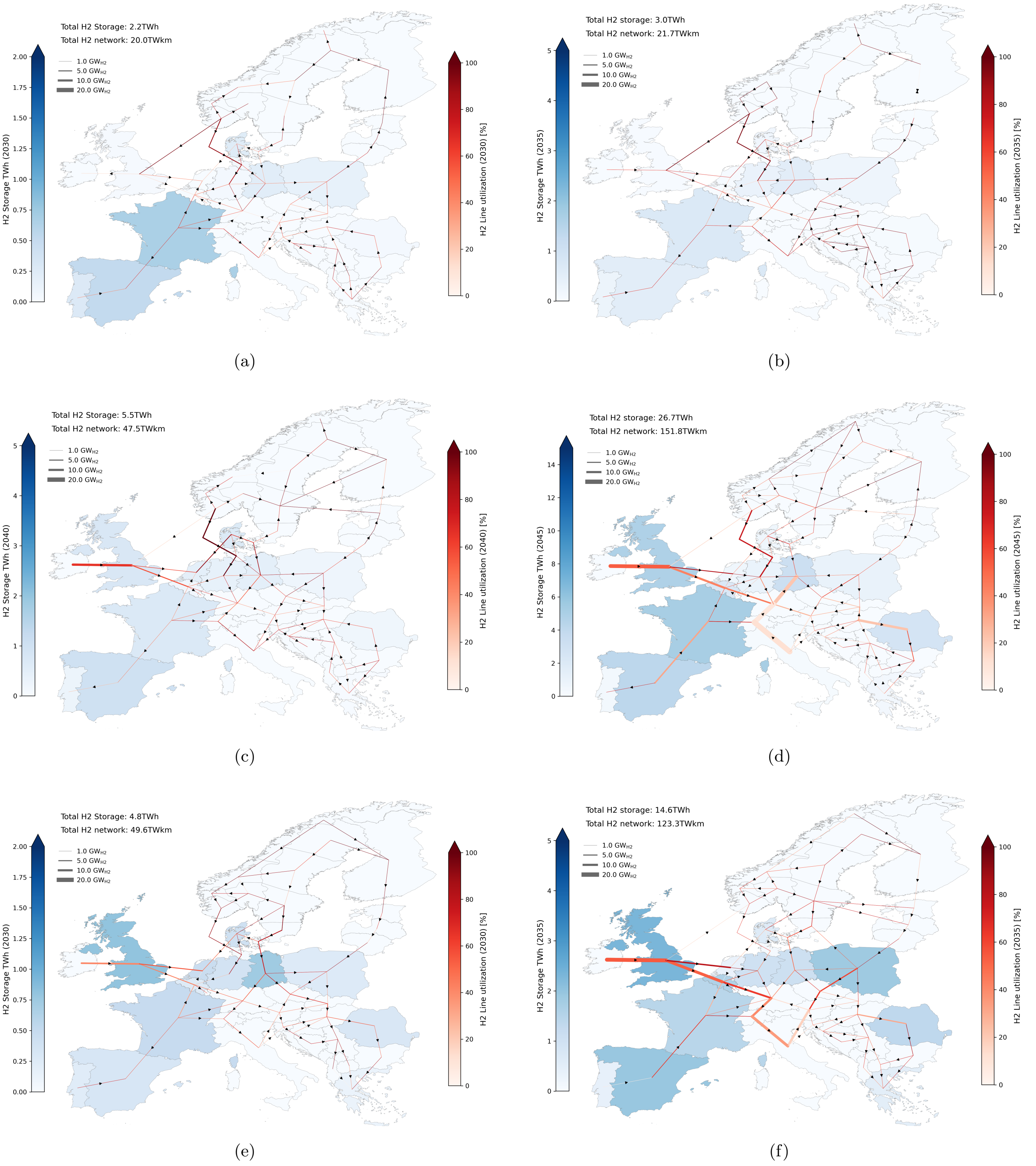

Supplementary Fig. 14: Optimised European hydrogen infrastructure and hydrogen trading. **a-d** Hydrogen Europe (H2E) scenario. **a**, 2030 **b**, 2035 **c**, 2040 **d**, 2045. The 2050 figure for H2E is displayed in the main manuscript. **e-f** Green Hydrogen Europe (GH2E) scenario. **e**, 2030 **f** 2035.

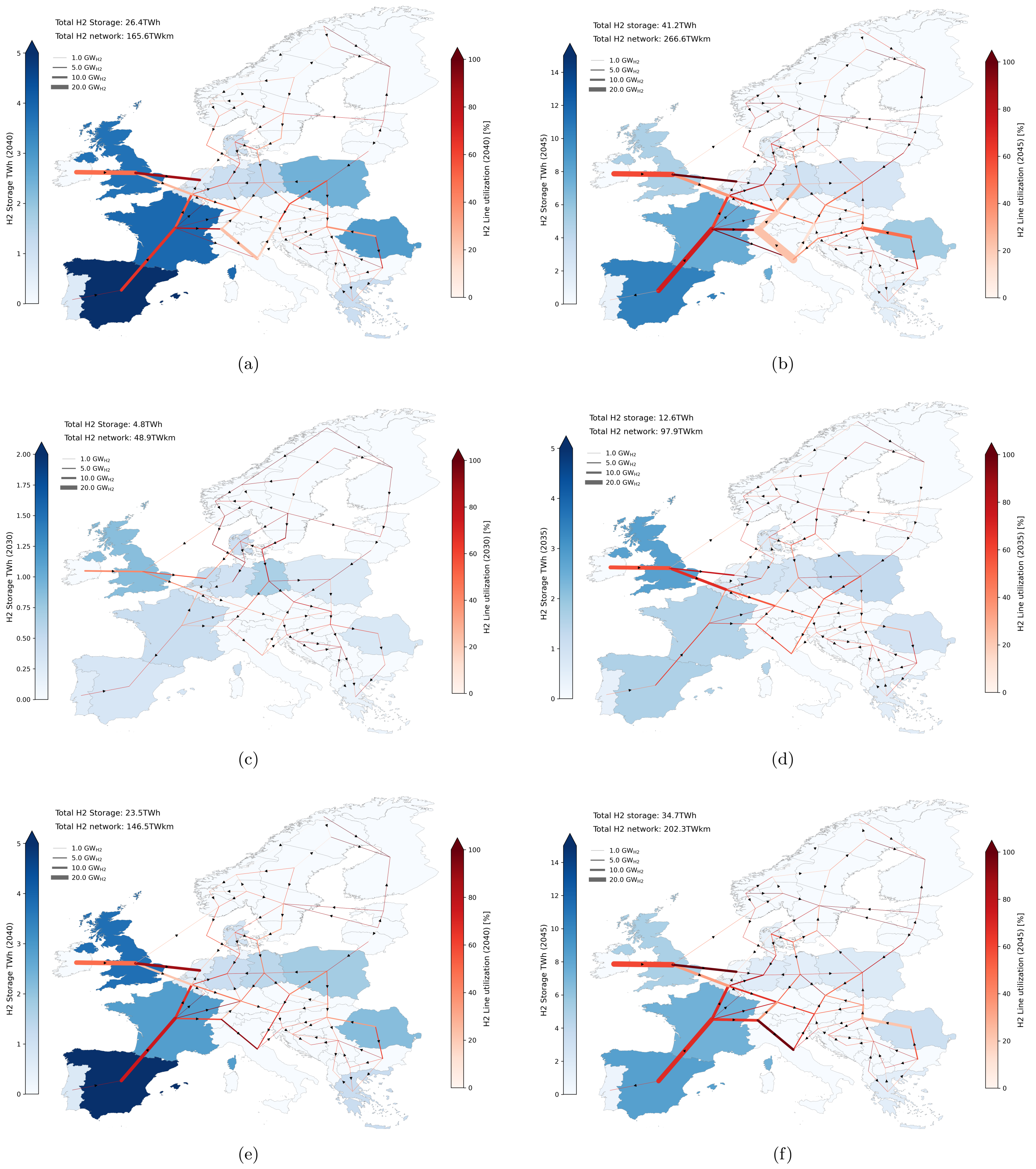

Supplementary Fig. 15: Optimised European hydrogen infrastructure and hydrogen trading. **a-b** Green Hydrogen Europe (GH2E) scenario. **a**, 2040 **b**, 2045 **c-f** Self-Sufficient Green Hydrogen Europe (SSGH2E) scenario. **c**, 2030 **d**, 2035 **e**, 2040 **f** 2045. The 2050 figure for GH2E and SSGH2E are displayed in the main manuscript.

Supplementary Discussion 1

Comparison to related studies: Renewable investments

The recent 2022 EU market outlook for solar power [39] reveals a significant surge of nearly 50% percent year-over-year in 2022, a record of 41.4 GW solar PV additional generation capacity. This development indicates that our near-term projections for 2030 (H2E scenario 55 GW/a) could be achieved, assuming similar increasing growth rates. However, our projections diverge from the European Commission's offshore wind power generation vision, which seeks to establish 300 GW of capacity by 2050 [40]. In the H2E scenario, we find 153 GW, whereas, in the GH2E scenario, a future system without blue hydrogen requires 213 GW offshore wind. When compared to other European system studies, a similar high renewable penetration is discovered. For example, Neumann et al. [41], by 2050, project solar PV installations ranging from 2666 to 3598 GW, onshore wind from 1691 to 1776 GW, and offshore wind from 206 to 245 GW. Another study [24] assessing 2050 carbon neutrality scenarios estimates solar PV ranges from 2146 to 2449 GW, onshore from 639 to 726 GW, and offshore from 201 to 227 GW.

Comparison to related studies: Blue hydrogen lock-in effect

In this study, variable renewable installations per region can reach a technical limit (see Supplementary Method 8, resource grades), implying that large-scale and deep electrification of the power, heat, and hydrogen sectors simultaneously may not be as efficient and cost-effective as planned. Utilizing locations where renewable resources exhibit high full-load hours and competitive investment costs could be used initially to electrify sectors where alternative technologies have high technological costs. Blue hydrogen lock-in investments reduce the requirement for additional power generation by 2035 and 2040 (Supplementary Fig. 7) while relying on falling natural gas prices and high anticipated emission capture rates of 90 %. In the H2E scenario, we project that 1444 TWh of natural gas is consumed overall across all sectors by 2050 when coupled with carbon capture applications. Nonetheless, the amount of natural gas needed to produce blue hydrogen is estimated to be 947 TWh, approximately 65 % of the total final natural gas demand. According to Eurostat [42], domestic natural gas extraction in the European Union (27 nations) in 2020 was 479 TWh, with Norway, accounting for another 1.140 TWh. Assuming that it is possible to sustain similar production rates in the future, producing blue hydrogen domestically by relying only on conventional fuels sourced from the EU nations and Norway is possible.

Seck et al. [43], using a European energy system model, emphasize the role of blue hydrogen, estimating a 20-52 % penetration by 2050, depending on scenario conditions. In addition, the Seck et al. illustrate that blue hydrogen will be more competitive than green in 2030. These results are aligned with the findings of our H2E scenario. Shirizadeh et al. [44] reveal in their global hydrogen study that blue hydrogen's prospects are limited under low electrolysis investment and favorable financial conditions. Our analysis, aligning with potential electrolysis investment trends, supports this outcome.

Finally, another recent energy system analysis study [45] examining the 2050 European electricity and hydrogen mix concludes that blue hydrogen may supply more than 60 % of the final hydrogen domestic demand, assuming capture rates of 89 % and a CO_2 tax by 2050 of 250 euro/ton similar to this study. However, blue hydrogen lock-in effect is dynamic, as examined in the Main Manuscript, section-Impact of tech, costs, and market dynamics on hydrogen paths can impact the hydrogen production pathway and Supplementary Notes 2 and 3.

Comparison to related studies: Hydrogen production and infrastructure

By comparing our electrolysis capacity growth to a recent study by Odenweller et al. [46], we illustrate that our projections for 2030 and 2050 are feasible in the scenarios. In addition, we emphasize that the SSGH2E scenario is consistent with the European Union's Hydrogen strategy (500 GW [46]) by 2050. We witness an electrolysis fleet with an installed capacity of 507 GW, which supplies a total hydrogen production of 1767 TWh. Another recent study [47] illustrates plausible pathways, towards 2050, for electrolysis deployment in the European system ranging from 1378 GW to 2186 GW, satisfying a total hydrogen demand of approximately 3150TWh. However, this demand is nearly twice as high as indicated by this study. According to the latest IEA Global Hydrogen Review 2023 [48], the current capacity of the European electrolysis fleet stands at approximately 0.5 GW. However, ambitious targets exceeding 60 GW by 2030 [49] have been announced in the RePowerEU [50] initiative. Our 2030 results range from 24-68 GW depending on the scenario.

Furthermore, we find no hydrogen imports from non-European nations by 2030 and moderate hydrogen imports by 2050 of around 7 % of the total demand. Nonetheless, the sensitivity analysis (Extended Data Fig. 6, main article) indicates that hydrogen imports could be increased if natural gas prices rise in the future or if electrolysis capital expenditures do not decrease substantially. Seck et al. [43] demonstrate similar results, highlighting zero imports of hydrogen by 2030 and 10-15 % of total demand in 2050.

Neumann et al. [41] utilize a detailed optimization approach and investigate the potential benefits of a European hydrogen network by 2050. However, the study disregards pathway dependencies and concludes that blue hydrogen plays a marginal role in the future energy system. The study demonstrates the need for a hydrogen network of 204–307 TWkm and 23-43 TWh of hydrogen storage. The results of the SSGH2E scenario for hydrogen infrastructure development of networks and storage are consistent with these findings. However, we illustrate the need for a hydrogen corridor from Southern Europe towards 2050. This difference may occur due to the possibility of storing underground hydrogen in Romania, which the above study may not consider. A scenario where hydrogen networks are not allowed, like Neumann et al., results in a similar magnitude of total system cost differences by 2050 of +1.49 % and +0.95 % for GH2E and H2E, respectively. In addition, Wetzel et al. [51] show a comparable topology of electrolysis production centers to the southern and western periphery of Europe by the year 2050, similar to the GH2E scenario (allowing hydrogen imports and green hydrogen).

Supplementary Note 1

Limitations

Several limitations of the analysis should be acknowledged. First, for computational efficiency, we restrict the spatial resolution of the model to large geographic regions in order to represent constraints between electricity market bidding zones, mostly covering entire countries. The nonlinear dynamics of electricity and gas flow are disregarded due to the absence of a granular distribution and transmission network model. Instead, we model an aggregated cross-regional interconnection capacities network. Second, we provide the option of storing hydrogen in large subterranean salt caverns or pressurized steel tanks. Notably, a potential degraded integrity of caprock in onshore underground hydrogen storage may lead to the contamination of underground water sources [52], limiting the possibility of onshore hydrogen storage. There is ongoing research into the prospective repurposing of depleted gas reserves in nations where salt caverns or aquifers are not available, but the technology readiness level is low [53, 54]. Third, the current study implicitly models CO_2 transport and storage. Future research should explore the integration of CO_2 network grids and storage within European contexts [55], yet through a sector-coupled approach. This approach could reveal additional benefits, notably in the production of renewable biogenic liquid fuels, especially considering regions with sustainable biomass potential. Fourth, we perform a sensitivity analysis to capture the effects of importing hydrogen derivatives such as ammonia, methanol, liquid hydrogen, and other liquid organic hydrogen carriers (LOHC). Future studies should evaluate the competition between various importing fuels via dedicated port terminals, transportation and storing options, and domestic European production. Noted that currently only liquid ammonia and methanol are competitive for importing without the need for re-conversion [11]. Finally, the study does not encompass the complete societal implications of green, blue, or imported hydrogen, including factors such as employment and public acceptance, which may have extensive and noteworthy effects [56]. The asymmetry of distributed energy investments in our analysis, such as extended PV investments in Spain and France, may have an impact on the level of social acceptance, possibly hindering the transition to a low-carbon energy system.

Supplementary Note 2

Combined impact of CO_2 capture rate, transport and storage costs

We perform a combined sensitivity analysis to evaluate the deployment of blue hydrogen in the H2E scenarios. We make the most pessimistic assumptions on transportation and storing cost of 40 €$_{2022}$/tCO_2 (approximately 100% increased from the base assumption of 20 €$_{2022}$/tCO_2) and vary the capture rate from 60% to 90%. Figure **??** shows that blue hydrogen share in the future system decreases as costs for managing CO_2 may change. We observe an early penetration of green hydrogen by 2030, accounting for 78% of the hydrogen market share in case of low capture rates

of 60%, with blue hydrogen facing out by 2050. However, we note that even with these pessimistic assumptions, blue hydrogen appears to be a pivotal energy carrier in the intermediate years, complementing green and hydrogen import options. Furthermore, our main conclusion that blue hydrogen remains an attractive solution for countries with low renewable potential installations is supported by looking at the spatial-temporal development of production centers and networks. For example, Italy or the northern industrial regions of Germany depend on blue hydrogen until 2050 (Supplementary Fig. 18), when it finally phases out.

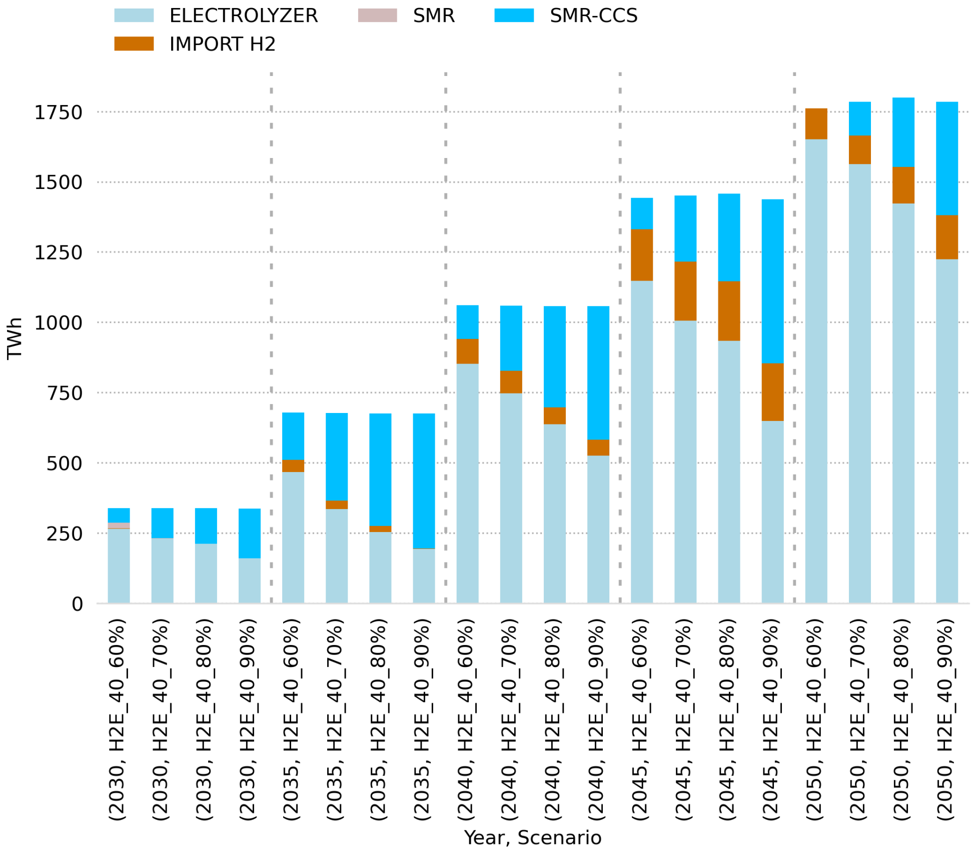

Supplementary Fig. 16: Sensitivity analysis of Hydrogen Europe (H2E) scenario hydrogen production pathway. Varying the CO_2 capture rate {60%, 90%} and assuming higher costs for transporting and storing CO_2, equal to 40 €/tCO_2. In the H2E scenario the costs are assumed equal to 20 €/tCO_2 .

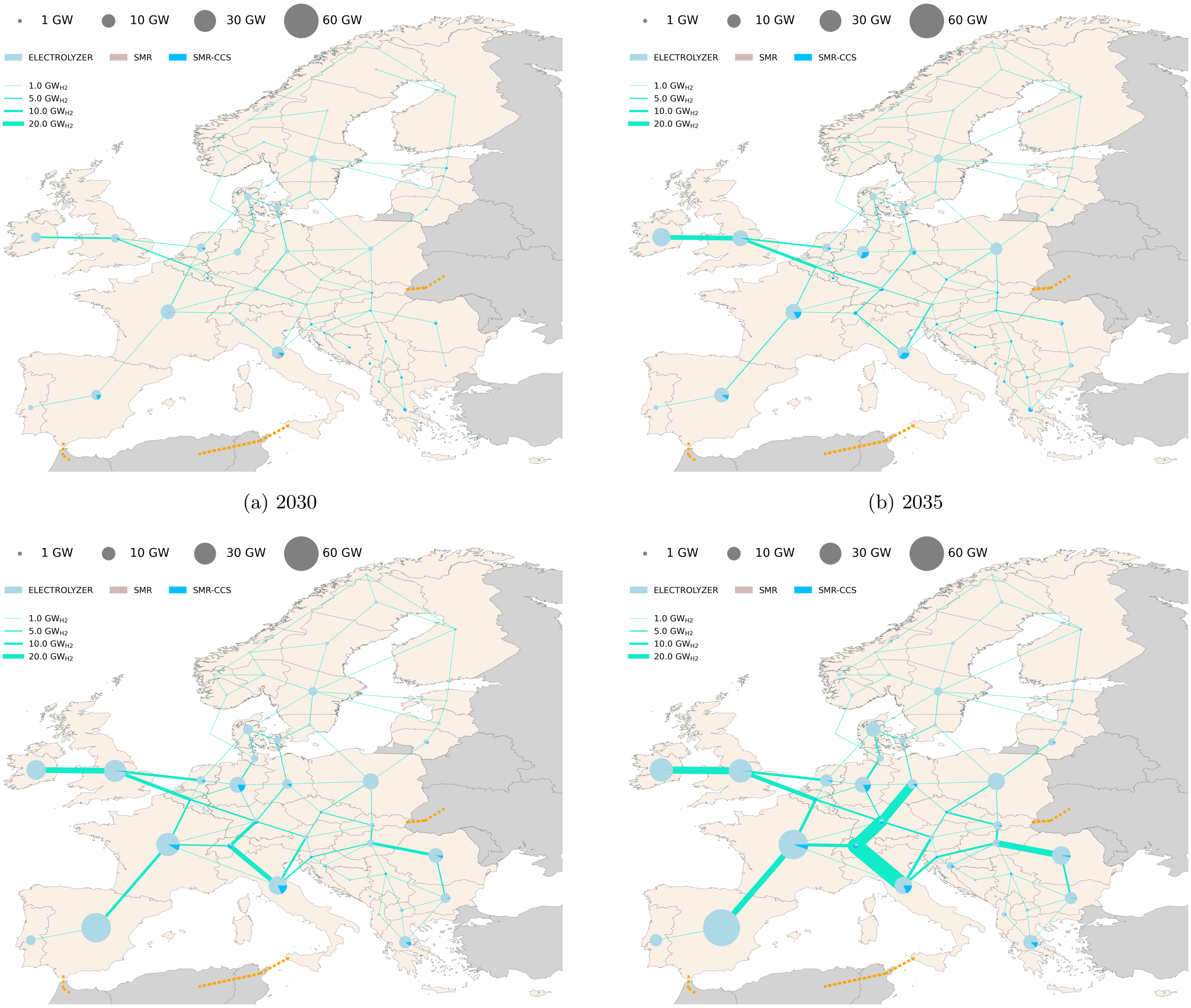

(a) 2030

(b) 2035

(c) 2040

(d) 2045

Supplementary Fig. 17: Hydrogen-optimized grid and production centers. Hydrogen Europe (H2E) scenario, assuming 60% CO_2 capture rate and 40 €$_{2022}$/tCO_2 for storing and transport. **a**, 2030. **b**, 2035. **c**, 2040. **d**, 2045.

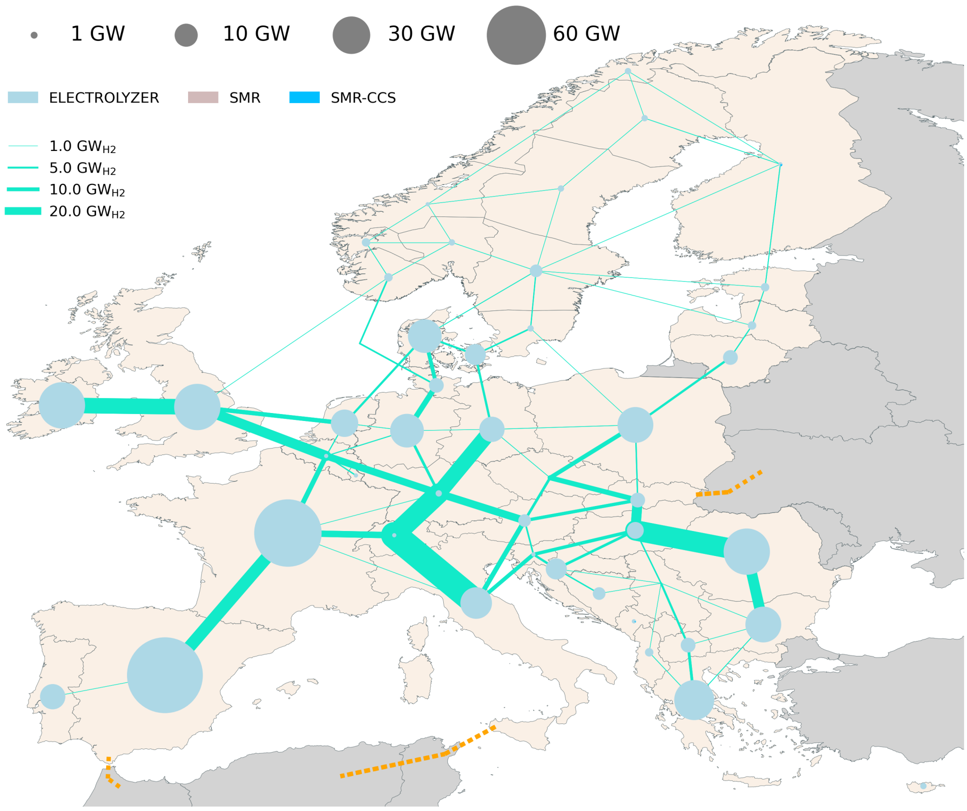

Supplementary Fig. 18: Hydrogen-optimized grid and production centers. Hydrogen Europe (H2E) scenario, assuming 60% CO_2 capture rate and 40 €/tCO_2 for storing and transport, Year: 2050.

Supplementary Note 3

Limit build-out of carbon capture and storage

The potential for CO_2 underground storage in Europe is estimated to be 625 Gt [57]. According to a recent study [58], the announced projects are anticipated to have a storage capacity of 172.83 Mt by 2030. Unlike salt caverns, CO_2 storage potential is spatially available in most European countries [59]. Supplementary Table 10 summarizes CO_2 capture and storage build-out on a system level. Considering the announced projects from 2025 to 2030 [58], we perform a linear regression to estimate the prospective evolution of CO_2 storage projects build-out, which can be found in Supplementary Table 10. It demonstrates that the model deployment of CCS technologies can be potentially realized.

Supplementary Table: 10: Carbon Captured and stored at System level, Hydrogen Europe (H2E) scenario. Announced projects [58] and future projection of CO_2 storage. Units: CO_2 Mt.

	2030	2035	2040	2045	2050
H2E	124.96	198.51	242.06	254.80	254.83
Projects	172.83	314.22	476.09	637.96	799.83

However, we note that in Balmorel, CCS investments are limited mainly to natural gas-based electricity, heat, and hydrogen production. Consequently, CCS for nonindustrial applications such as bioenergy and direct air capture and other industrial applications are not considered. In the future, various CCS projects will compete for the CO_2 storage availability of the announced storage project, potentially limiting blue hydrogen production. To evaluate potential effects, we perform a sensitivity analysis where we limit on a system level the potential CO_2 storage based on the data generated in Supplementary Table 10 and incrementally reduce the potential.

Supplementary Fig. 19 depicts that limiting potential CO_2 storage availability at a system level promotes domestic green hydrogen and imports to meet final demand. The build-out constraint is binding mainly on the initial years (2020-2040), affecting the 2050 results only if restricted assumptions are met. Assuming a limited 20 % storage potential leads to a 35 % blue hydrogen production decrease by 2050.

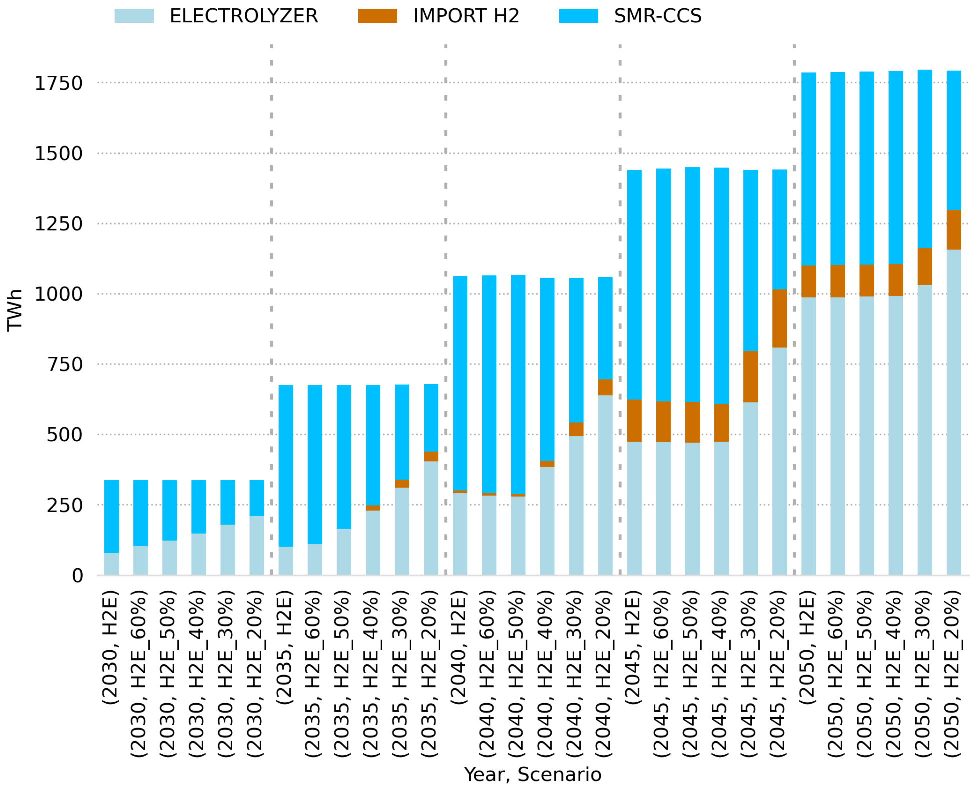

Supplementary Fig. 19: Sensitivity analysis of Hydrogen Europe (H2E) scenario hydrogen production pathway. Varying the CO_2 storage total system potential [60%, 40%, 30%, 20%]. For the system total potential see Supplementary Table 10 - Projects.

Supplementary Note 4

Importing of ammonia and high-value chemicals

Currently, there are proposals for the establishment of import terminals for hydrogen derivatives (see Supplementary Method 5) in Europe. Our sensitivity analysis investigates the impact on the hydrogen network and storage when ammonia and high-value chemicals are imported. The sensitivity analysis is carried out by gradually decreasing the exogenous demand for those fuels (see Supplementary Table 4) from 10 % (84 TWh by 2050) to 60 % (500 TWh by 2050) assuming shipping imports. The sensitivity analysis is conducted on both the H2E and GH2E scenarios to account for the impact of blue hydrogen.

As Supplementary Figures 20a - 20b show, substituting domestic ammonia and high-value chemicals hydrogen demand with overseas imports, decreases overall storage and network investment. By 2035 and 2040, in the H2E scenario, the effect of

shipping imports is minimal due to the presence of blue hydrogen serving as a baseload production technology, satisfying the overall lower domestic hydrogen demand. On the contrary, in the GH2E scenario, shipping imports can have a significant impact on the requirement for European infrastructure development due to the intermediate production of green hydrogen. For example, in 2035, the storage and network expansion decreased by 51 % (Supplementary Fig. 21a) and 49 % (Supplementary Fig. 21b), respectively, for a 60 % shipping import scenario. Looking further into the future, in the 2050 H2E scenario, 84TWh shipping imports result in a 6 % network relative change, while 500TWh imports result in a 42 % (Supplementary Fig. 20b). In the GH2E scenario, the network relative reduction is lower by approximately 25 %, and the underground storage is decreased by 30 %. Regarding the spatial production of hydrogen, shipping imports highly affect the solar-rich production centers such as France and Spain (Supplementary Figures 22a - 23b), and overall decrease the need of renewable assets deployment.

It should be noted that the current sensitivity analysis investigates the influence of hydrogen shipping imports on the future development of European hydrogen infrastructure. However, it does not examine the competitiveness of shipping with pipeline imports, nor does it shed light on the competition of domestically generated hydrogen derivatives with shipping import alternatives.

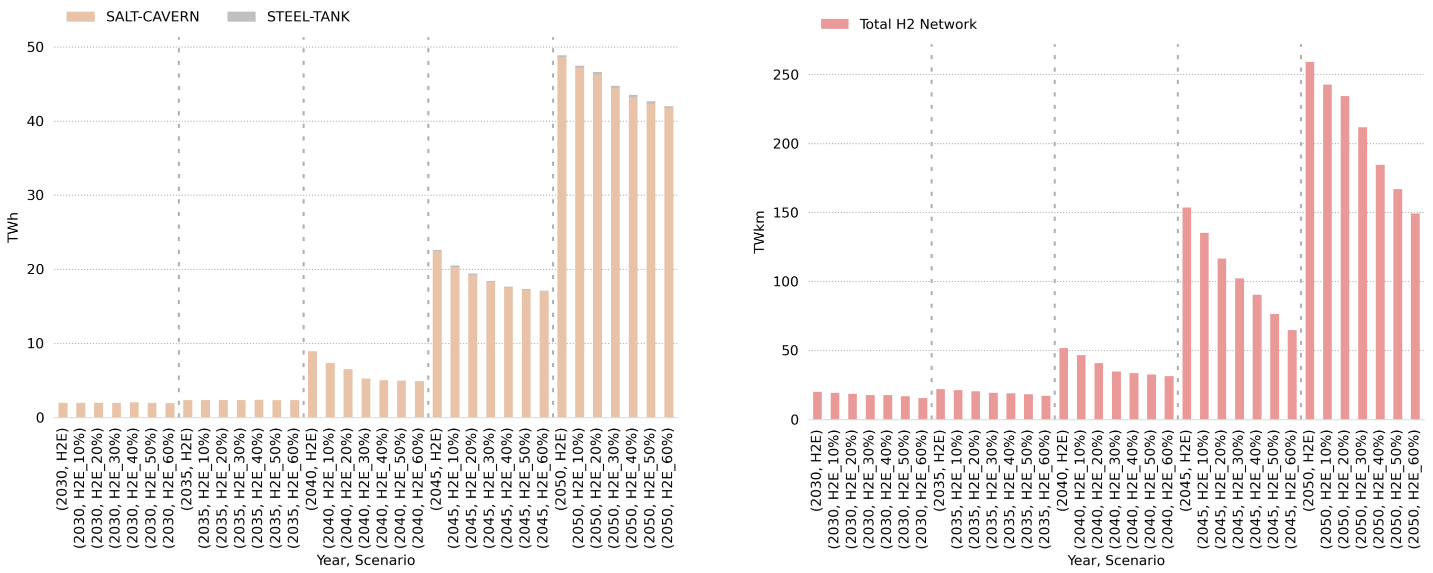

(a) (b)

Supplementary Fig. 20: Ammonia and synthetic fuels import sensitivity analysis. Hydrogen Europe (H2E) scenario: **a**, Hydrogen storage investments. **b**, Total hydrogen network development.

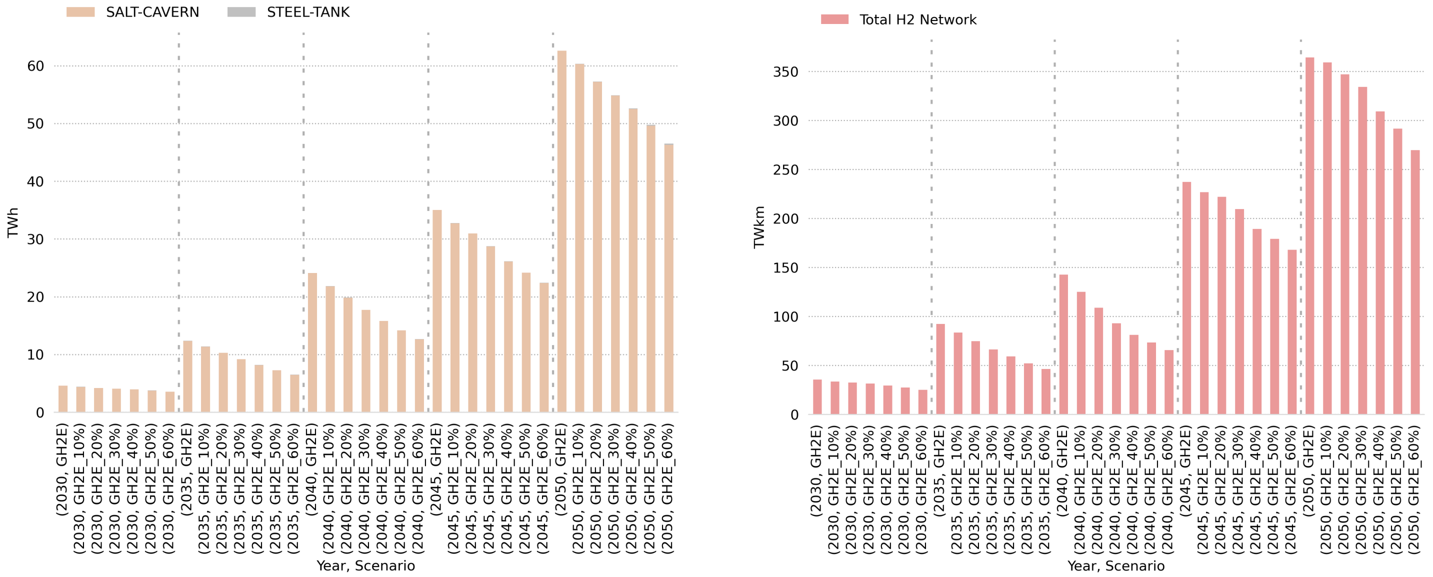

(a) (b)

Supplementary Fig. 21: Ammonia and synthetic fuels import sensitivity analysis. Green Hydrogen Europe (GH2E) scenario: **a**, Hydrogen storage investments. **b**, Total hydrogen network development.

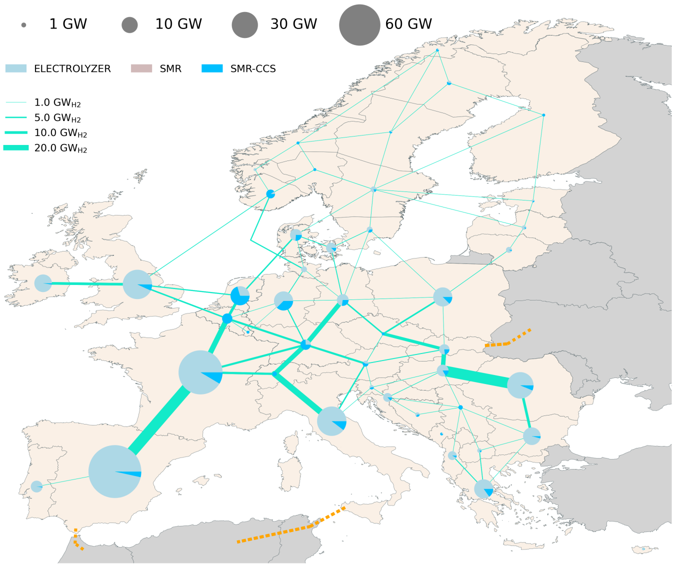

(a) (b)

Supplementary Fig. 22: Hydrogen-optimized grid and production centers. Hydrogen Europe (H2E) scenario 2050: **a**, 10% (84 TWh) Imports. **b**, 60% (500 TWh) Imports.

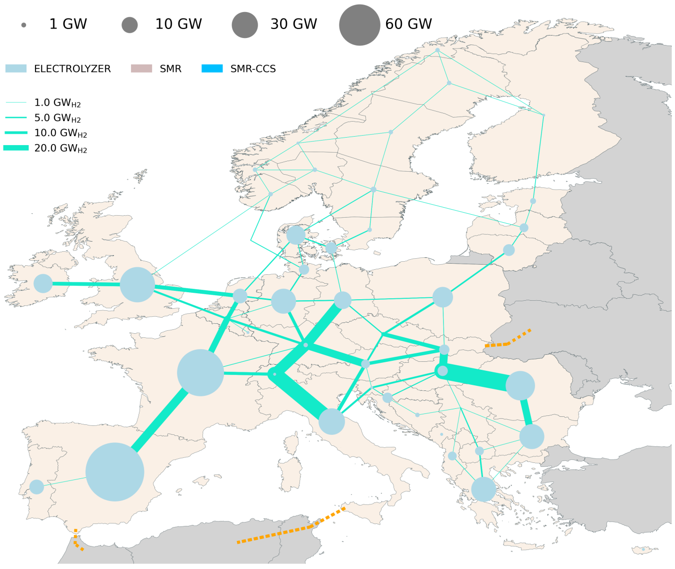

(a) (b)

Supplementary Fig. 23: Hydrogen-optimized grid and production centers. Green Hydrogen Europe (GH2E) scenario 2050: **a**, 10% (84 TWh) Imports. **b**, 60% (500 TWh) Imports.

Supplementary Note 5

Ammonia and high-value chemicals demand allocation sensitivity

Here, we question if the uncertain spatial demand allocation for ammonia and high-value chemicals (HVC) could potentially be shifted to places where competitive hydrogen production occurs. A sensitivity analysis on the exogenous demand allocation of hydrogen derivatives demand is applied to evaluate the robustness of the hydrogen network development as depicted in scenarios H2E and GH2E. However, we allow only the ammonia and HVC demand to shift since it is expected that the production of those fuels will be co-located with hydrogen production in regional hubs such as ports or industrial clusters [49]. The methodology applied can be found in section **??**.

Supplementary Fig. 24 demonstrates the optimal demand distribution under the assumption that each Balmorel region possesses a 60 % spatial shift flexibility ($\theta = 0.6$). The exogenous European system level demand for ammonia and HVC is estimated to be equal to 1023 TWh by 2050 [33], suggesting that a total of 613 TWh is able to shift between the model regions. Across the two scenarios, we observe similar resulting trends. The large production centers located at the periphery of Europe attract significant demand for hydrogen derivatives (Supplementary Fig. 25). Furthermore, industrial regions of Germany and nations such as Belgium and the Netherlands fully utilize the available shifting potential, relaxing the network development expansion requirements as captured in Supplementary Figures 26a and 26b. The corridors from the United Kingdom and Ireland or from Spain and France appear significantly smaller. In addition, a sensitivity analysis of the flexibility parameter θ reveals that demand allocation can significantly impact the European network, particularly if blue hydrogen is unavailable (Supplementary Figures 27a and 27b). For example, in the case of the GH2E scenario, a 60 % available shift could result in a 18 % lower Pan-European network development by 2050, while a 100 % shift results in a 26 % decrease.

These results illustrate the benefits of regional co-production of hydrogen and derivatives as they can decrease the need for cross-border hydrogen connections, altering the shape of the visioned European Hydrogen Backbone reports [9]. It should be noted that, due to relative uncertainty, the additional transportation costs of the derivative to the final consumption location are not accounted for. We take the opportunity to discuss that the current methodology could be expanded to a general investigation of future European industrial demand relocation, reducing the need for cross-border infrastructure development. Further costs for relocating existing production centers and transportation expenses must be accounted for. Furthermore, social and macroeconomic indicators, as well as national industry policies, should also be assessed to provide additional insights into the potential benefits.

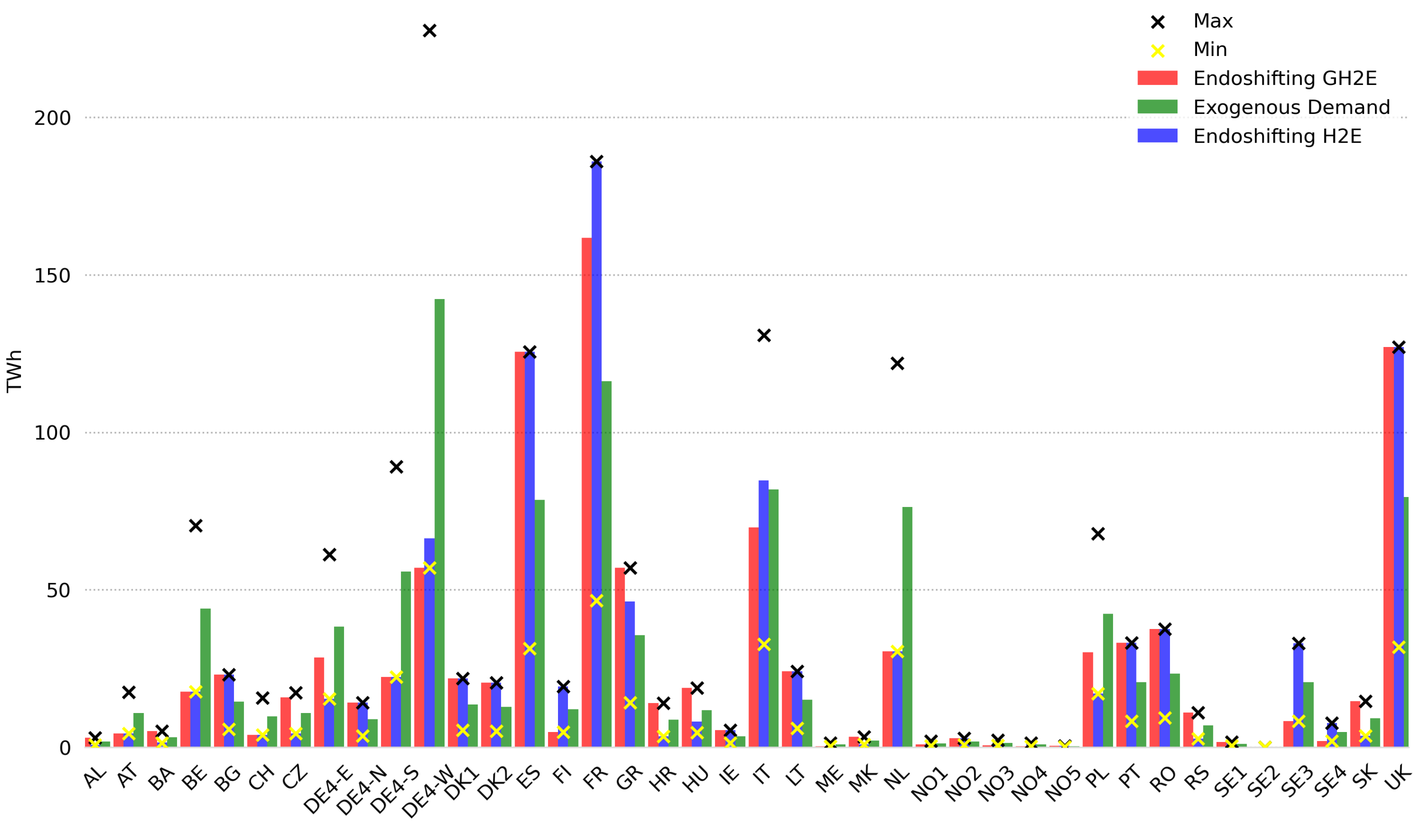

Supplementary Fig. 24: Ammonia and high-value chemicals endogenous demand shift, allowed flexibility $\theta = 0.6$, both Hydrogen Europe (H2E) and Green Hydrogen Europe (GH2E) scenarios. The x icon illustrates the maximum and minimum potential. Year 2050.

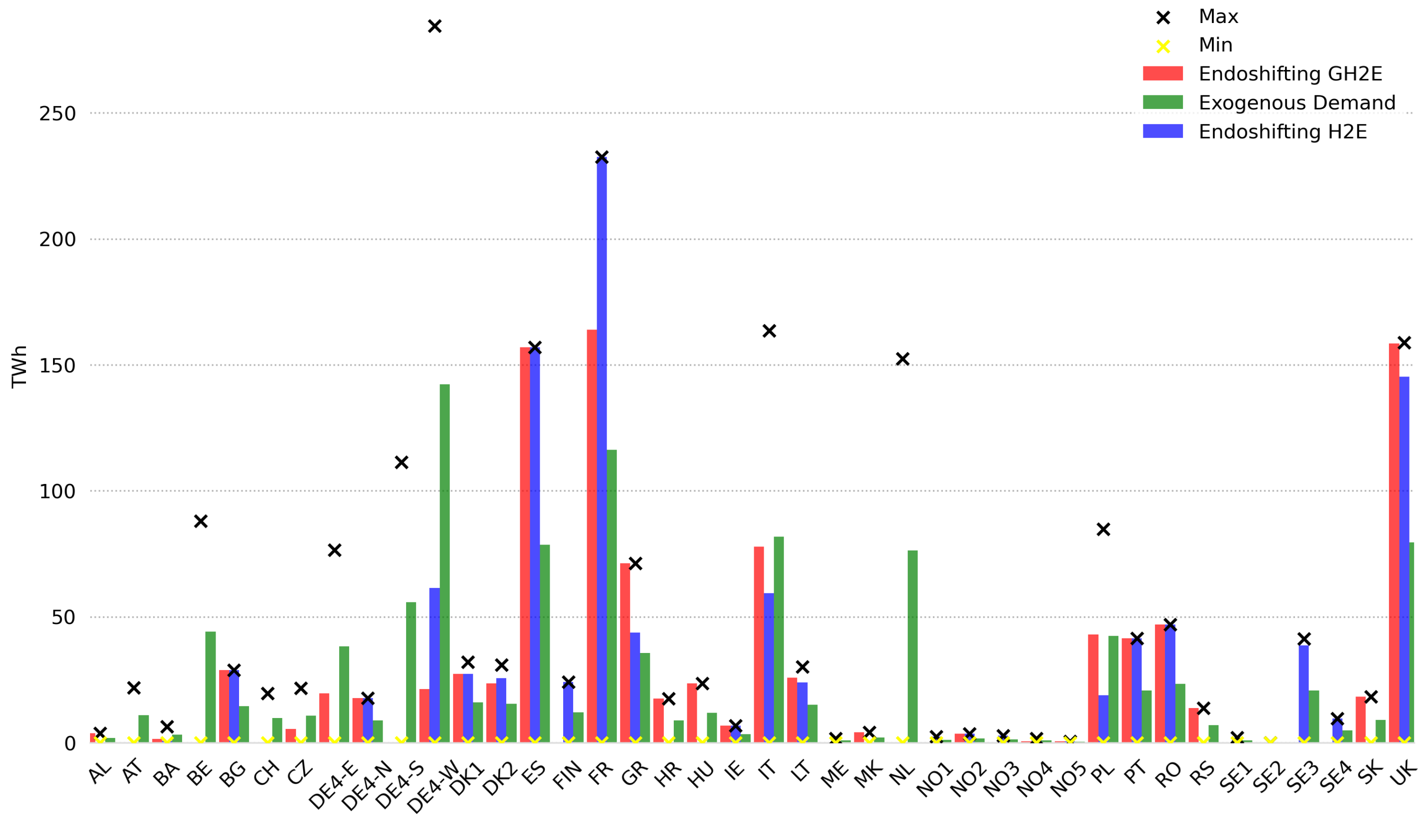

Supplementary Fig. 25: Ammonia and high-value chemicals endogenous demand shift, allowed flexibility $\theta = 1$, both Hydrogen Europe (H2E) and Green Hydrogen Europe (GH2E) scenarios. The x icon illustrates the maximum and minimum potential. Year 2050.

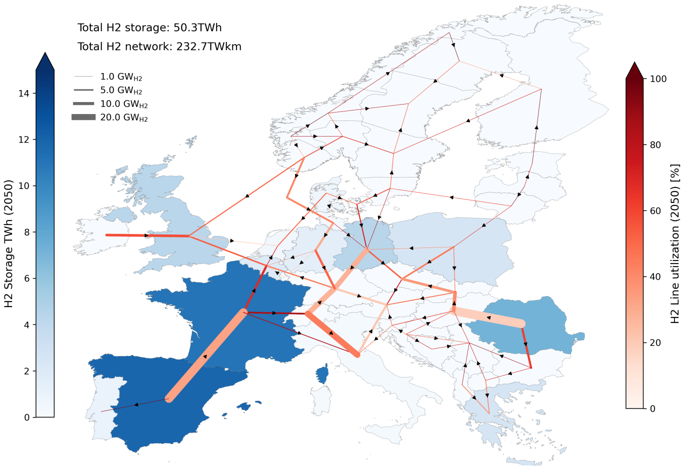

(a)

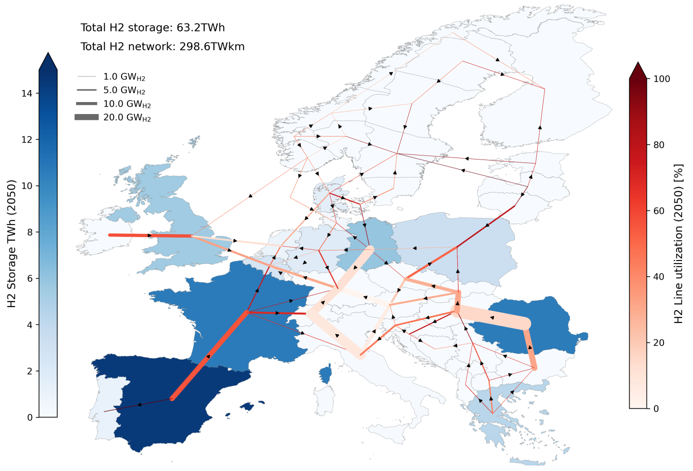

(b)

Supplementary Fig. 26: Optimised European hydrogen infrastructure and hydrogen trading by 2050, Ammonia and HVC fuels endogenous demand sensitivity, $\theta = 0.6$. **a**, Hydrogen Europe (H2E) scenario **b**, Green Hydrogen Europe (GH2E) scenario.

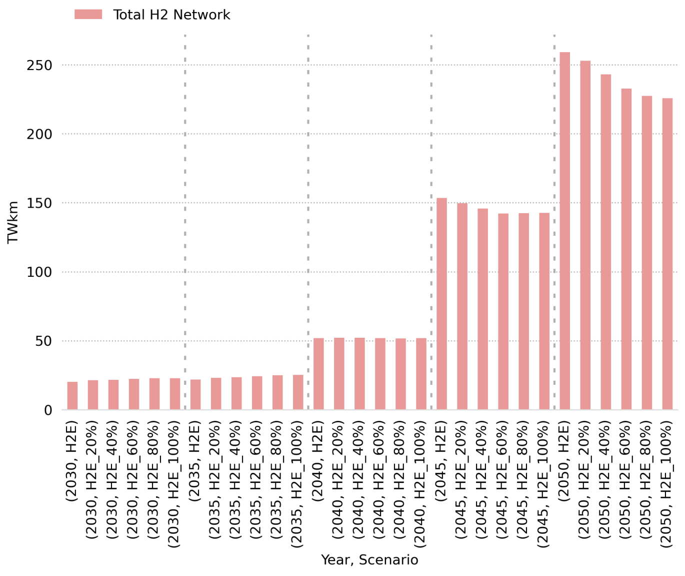

(a)

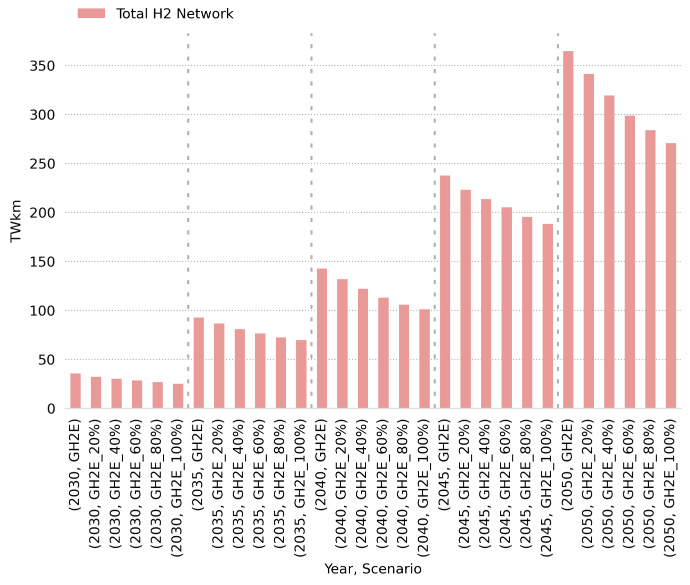

(b)

Supplementary Fig. 27: Ammonia and HVC fuels endogenous demand sensitivity on the hydrogen network development. **a**, Hydrogen Europe (H2E) scenario **b**, Green Hydrogen Europe (GH2E) scenario.

Supplementary Note 6

CO_2 quotas

Following the Net Zero Emissions by 2050 (NZE) scenario, the CO_2 quota projections (see Supplementary Table 11) are extracted from the World Energy Outlook (WEO 2022) [60]. The trend relates to countries within the Organisation for Economic Cooperation and Development (OECD).

Supplementary Table: 11: CO2 projections based on World Energy Outlook (WEO 2022) [60]

CO_2 quotas (€$_{2016}$/ton)						
2020	2025	2030	2035	2040	2045	2050
78	109	140	173	205	228	250

Supplementary Note 7

Fuel prices

The fuel prices reflect the assumptions of the WEO (2022) scenario Net Zero Emissions by 2050 [60]. We observe that the most recent iteration of WOE has revised its price forecasts, particularly for natural gas and coal, to account for the disruption of the European energy market. The scenario implies that fossil fuel demand will decrease as a result of the increasing CO_2 price taxation (see Supplementary Table 11). Consequently, fossil fuel prices (Supplementary Table 12) will decline sharply until 2030 before reaching a plateau.

Supplementary Table: 12: Fuel price trajectory, data extracted from World Energy Outlook (WEO 2022) [60] and [2]

€$_{2016}/GJ$	2020	2025	2030	2035	2040	2045	2050
Nuclear	0.76	0.76	0.76	0.76	0.76	0.76	0.76
Natural gas	5.83	8.00	3.66	3.50	3.34	3.18	3.03
Coal	3.66	2.57	1.49	1.42	1.35	1.28	1.20
Lignite	0.75	0.88	1.01	1.00	0.99	0.98	0.96
Fuel oil	5.43	8.76	12.10	11.96	11.82	11.68	11.54
Heavy fuel oil	12.60	12.60	12.60	12.60	12.60	12.60	12.60
Straw	5.17	6.16	7.16	8.06	8.96	9.23	9.51
Wood waste	0.65	0.65	0.65	0.65	0.65	0.65	0.65
Wood chips	6.20	6.20	6.20	6.20	6.20	6.20	6.20
Wood pellets	8.65	9.65	10.65	11.55	12.44	12.72	12.99
Biogas	12.72	12.72	12.72	12.72	12.72	12.72	12.72
LNG	7.64	10.36	11.27	11.93	12.58	13.24	13.89

Supplementary Note 8

Technology assumptions

The technology data (see Supplementaty Table 13) are mainly extracted from the Danish Energy Agency (DEA) technology Catalogues [3]. All the financial data is given in €$_{2016}$ level.

Supplementary Table: 13: Technology assumptions

Name	Parameter	2020	2030	2040	2050	Unit	Source
Biogas internal combustion backpressure	Fuel efficiency	0.930	0.939	0.960	0.940	0-1	[3]
	Cb	0.86	0.92	0.92	1	-	[3]
	Investment cost	0.931	0.882	0.858	0.833	M€/MW	[3]
	FOM	0.860	0.920	0.920	1.000	k€/MW	[3]
	VOM	3.161	3.087	2.930	2.764	€/MWh	[3]
	Economic lifetime	25	25	25	25	years	[3]
Biogas internal combustion condensing	Fuel efficiency	0.430	0.450	0.460	0.470	0-1	[3]
	Investment cost	0.791	0.750	0.729	0.708	M€/MW	[3]
	FOM	9.555	9.114	8.722	8.330	k€/MW	[3]
	VOM	7.350	6.860	6.370	5.880	€/MWh	[3]
	Economic lifetime	25	25	25	25	years	[3]
Coal steam Supercritical condensing	Fuel efficiency	0.49	0.52	0.53	0.54	0-1	[3]
	Investment cost	1.69099	1.65767	1.61602	1.57437	M€/MW	[3]
	FOM	60.368	60.368	60.368	60.368	k€/MW	[3]
	VOM	2.156	2.156	2.156	2.156	€/MWh	[3]
	Economic Lifetime	40	40	40	40	years	[3]
Coal steam Supercritical extraction	Fuel efficiency	0.49	0.52	0.53	0.54	0-1	[3]
	Cb	0.84	1.01	1.01	1.01	-	[3]
	Cv	0.15	0.15	0.15	0.15	-	[3]
	Investment cost	1.9894	1.9502	1.9012	1.8522	M€/MW	[3]
	FOM	60.368	60.368	60.368	60.368	k€/MW	[3]
	VOM	1.05644	1.12112	1.14268	1.16424	€/MWh	[3]
	Economic Lifetime	40	40	40	40	years	[3]
Electric boiler	Fuel efficiency	0.993	0.993	0.993	0.993	0-1	[3]
	Investment cost	0.388	0.370	0.352	0.334	M€/MW	[3]
	FOM	3.312	3.171	3.029	2.888	k€/MW	[3]
	VOM	0.588	0.653	0.653	0.653	€/MWh	[3]
	Economic Lifetime	25	25	25	25	years	[3]
Electric battery storage	Fuel efficiency	0.805	0.875	0.950	0.950	0-1	[3]
	Investment cost	0.304	0.213	0.247	0.164	M€/MW	[3]
	FOM	23.071	17.006	0.909	0.606	k€/MW	[3]
	VOM	0	0	0	0	€/MWh	[3]
	Economic Lifetime	20	20	20	20	years	[3]
Air to air heat pump	Fuel efficiency	4.9	4.9	4.9	4.9	0-1	[3]
	Investment cost	0.431	0.431	0.431	0.431	M€/MW	[3]
	FOM	63.374	41.774	39.782	37.789	k€/MW	[3]
	VOM	0	0	0	0	€/MWh	[3]
	Economic Lifetime	12	12	12	12	years	[3]
Air to water heat pump	Fuel efficiency	3.240	3.377	3.442	3.607	0-1	[3]
	Investment cost	1.212	1.068	0.990	0.912	M€/MW	[3]
	FOM	35.067	32.249	31.269	30.289	k€/MW	[3]
	VOM	1.012	0.910	0.885	0.860	€/MWh	[3]
	Economic Lifetime	22	22	25	25	years	[3]
Water to water heat pump	Fuel efficiency	6.000	7.600	7.600	10.467	0-1	[3]
	Investment cost	0.645	0.580	0.580	0.522	M€/MW	[3]
	FOM	1.960	1.960	1.960	1.960	k€/MW	[3]
	VOM	1.764	1.666	1.666	1.568	€/MWh	[3]
	Economic Lifetime	25	25	25	25	years	[3]
Ground water heat pump	Fuel efficiency	3.650	3.800	3.913	4.025	0-1	[3]
	Investment cost	1.056	0.975	0.911	0.848	M€/MW	[3]
	FOM	10.173	9.244	8.828	8.413	k€/MW	[3]
	VOM	0.882	0.833	0.809	0.784	€/MWh	[3]
	Economic Lifetime	25	25	25	25	years	[3]
Pump hydro reservoir	Fuel efficiency	0.800	0.800	0.800	0.800	0-1	[3]
	Investment cost	0.285	0.285	0.285	0.285	M€/MW	[3]
	FOM	0.735	0.735	0.735	0.735	k€/MW	[3]
	VOM	0.000	0.000	0.000	0.000	€/MWh	[3]
	Economic Lifetime	60	60	60	60	years	[3]
Pit heat storage seasonal	Fuel efficiency	0.7	0.7	0.7	0.7	0-1	[3]
	Investment cost	0.00094	0.00090	0.00085	0.00079	M€/MW	[3]
	FOM	0.00300	0.00300	0.00300	0.00300	k€/MW	[3]
	VOM	0	0	0	0	€/MWh	[3]
	Economic Lifetime	20	20	20	20	years	[3]
Watertank heat storage	Fuel efficiency	0.965	0.965	0.965	0.965	0-1	[3]
	Investment cost	0.20235	0.20235	0.20235	0.20235	M€/MW	[3]
	FOM	8.17088	8.17088	8.17088	8.17088	k€/MW	[3]
	VOM	0.343	0.343	0.343	0.343	€/MWh	[3]
	Economic Lifetime	35	35	35	35	years	[3]
Municipal waste heat only boiler	Fuel efficiency	1.060	1.060	1.060	1.060	0-1	[3]
	Investment cost	1.903	1.814	1.762	1.710	M€/MW	[3]
	FOM	79.765	74.764	71.291	67.818	k€/MW	[3]
	VOM	6.215	6.230	6.245	6.260	€/MWh	[3]
	Economic Lifetime	25	25	25	25	years	[3]

Municipal waste subcritical steam backpressure	Fuel efficiency	1.014	1.040	1.047	1.054	0-1	[3]
	Cb	0.293	0.300	0.300	0.300	-	[3]
	Investment cost	8.946	8.383	7.934	7.485	M€/MW	[3]
	FOM	261.333	241.733	225.400	209.067	k€/MW	[3]
	VOM	5.661	5.737	5.700	5.662	€/MWh	[3]
	Economic Lifetime	25	25	25	25	years	[3]
Municipal waste subcritical steam condensing	Fuel efficiency	0.230	0.240	0.242	0.243	0-1	[3]
	Investment cost	7.604	7.126	6.744	6.362	M€/MW	[3]
	FOM	261.333	241.733	225.400	209.067	k€/MW	[3]
	VOM	24.613	23.903	23.590	23.276	€/MWh	[3]
	Economic Lifetime	25	25	25	25	years	[3]
Natural gas heat only boiler	Fuel efficiency	0.987	0.992	0.996	1.002	0-1	[3]
	Investment cost	0.089	0.107	0.107	0.101	M€/MW	[3]
	FOM	4.011	5.390	3.557	4.880	k€/MW	[3]
	VOM	0.694	0.784	0.823	0.862	€/MWh	[3]
	Economic Lifetime	21.4286	24	24	24	years	[3]
Natural gas combined cycle backpressure	Fuel efficiency	0.902	0.909	0.926	0.905	0-1	[3]
	Cb	1.300	1.400	1.400	1.550	-	[3]
	Investment cost	1.880	1.735	1.663	1.590	M€/MW	[3]
	FOM	28.714	27.244	26.362	25.480	k€/MW	[3]
	VOM	2.199	2.181	2.170	2.156	€/MWh	[3]
	Economic Lifetime	25	25	25	25	years	[3]
Natural gas combined cycle condensing	Fuel efficiency	0.550	0.570	0.580	0.590	0-1	[3]
	Investment cost	1.340	1.247	1.207	1.168	M€/MW	[3]
	FOM	28.714	27.244	26.362	25.480	k€/MW	[3]
	VOM	4.312	4.116	4.018	3.920	€/MWh	[3]
	Economic Lifetime	25	25	25	25	years	[3]
Natural gas combined cycle extraction	Fuel efficiency	0.590	0.610	0.620	0.630	0-1	[3]
	Cb	1.800	2.000	2.000	2.200	-	[3]
	Cv	0.150	0.150	0.150	0.150	-	[3]
	Investment cost	1.272	1.200	1.178	1.157	M€/MW	[3]
	FOM	28.714	27.244	26.362	25.480	k€/MW	[3]
	VOM	2.544	2.511	2.491	2.470	€/MWh	[3]
	Economic Lifetime	25	25	25	25	years	[3]
Natural gas internal combustion backpressure	Fuel efficiency	0.965	0.965	0.985	0.981	0-1	[3]
	Cb	0.950	0.990	0.990	1.040	-	[3]
	Investment cost	1.374	1.301	1.265	1.229	M€/MW	[3]
	FOM	9.555	9.114	8.722	8.330	k€/MW	[3]
	VOM	2.487	2.399	2.401	2.401	€/MWh	[3]
	Economic Lifetime	25	25	25	25	years	[3]
Natural gas internal combustion condensing	Fuel efficiency	0.470	0.480	0.490	0.500	0-1	[3]
	Investment cost	1.168	1.106	1.075	1.045	M€/MW	[3]
	FOM	9.555	9.114	8.722	8.330	k€/MW	[3]
	VOM	5.292	4.998	4.900	4.802	€/MWh	[3]
	Economic Lifetime	25	25	25	25	years	[3]
Natural gas Turbine Generator backpressure	Fuel efficiency	0.811	0.842	0.852	0.862	0-1	[3]
	Cb	0.950	0.950	0.950	0.950	-	[3]
	Investment cost	0.954	0.911	0.889	0.868	M€/MW	[3]
	FOM	19.110	18.228	17.934	17.640	k€/MW	[3]
	VOM	1.885	1.860	1.813	1.764	€/MWh	[3]
	Economic Lifetime	25	25	25	25	years	[3]
Natural gas Turbine Generator condensing	Fuel efficiency	0.395	0.410	0.415	0.420	0-1	[3]
	Investment cost	0.811	0.774	0.756	0.737	M€/MW	[3]
	FOM	19.110	18.228	17.934	17.640	k€/MW	[3]
	VOM	4.802	4.557	4.386	4.214	€/MWh	[3]
	Economic Lifetime	25	25	25	25	years	[3]
Natural gas steam Subcritical Turbine backpressure	Fuel efficiency	0.763	0.763	0.763	0.763	0-1	[3]
	Cb	0.101	0.101	0.101	0.101	-	[3]
	Investment cost	0.868	0.868	0.868	0.868	M€/MW	[3]
	FOM	12.051	12.051	12.051	12.051	k€/MW	[3]
	VOM	0.318	0.318	0.318	0.318	€/MWh	[3]
	Economic Lifetime	30	30	30	30	years	[3]
Natural gas steam Subcritical Turbine condensing	Fuel efficiency	0.470	0.470	0.470	0.470	0-1	[3]
	Investment cost	0.909	0.909	0.909	0.909	M€/MW	[3]
	FOM	37.240	37.240	37.240	37.240	k€/MW	[3]
	VOM	0.804	0.804	0.804	0.804	€/MWh	[3]
	Economic Lifetime	30	30	30	30	years	[3]
Natural gas steam Subcritical Turbine extraction	Fuel efficiency	0.470	0.470	0.470	0.470	0-1	[3]
	Cb	0.700	0.700	0.700	0.700	-	[3]
	Cv	0.170	0.170	0.170	0.170	-	[3]
	Investment cost	1.069	1.069	1.069	1.069	M€/MW	[3]
	FOM	37.240	37.240	37.240	37.240	k€/MW	[3]
	VOM	0.378	0.378	0.378	0.378	€/MWh	[3]
	Economic Lifetime	30	30	30	30	years	[3]
Peat steam Subcritical Turbine backpressure	Fuel efficiency	1.151	1.151	1.151	1.151	0-1	[3]
	Cb	0.250	0.250	0.250	0.250	-	[3]
	Investment cost	4.788	4.547	4.368	4.190	M€/MW	[3]
	FOM	205.800	200.900	193.550	186.200	k€/MW	[3]
	VOM	1.083	1.081	1.086	1.091	€/MWh	[3]
	Economic Lifetime	25	25	25	25	years	[3]
Straw heat only boiler	Fuel efficiency	1.020	1.020	1.020	1.020	0-1	[3]
	Investment cost	0.872	0.823	0.784	0.745	M€/MW	[3]
	FOM	50.274	47.432	44.933	42.434	k€/MW	[3]
	VOM	0.588	0.588	0.588	0.588	€/MWh	[3]
	Economic Lifetime	25	25	25	25	years	[3]
Straw steam Subcritical Turbine backpressure	Fuel efficiency	0.956	0.956	0.956	0.956	0-1	[3]
	Cb	0.363	0.363	0.363	0.363	-	[3]
	Investment cost	3.779	3.614	3.442	3.270	M€/MW	[3]
	FOM	169.867	160.067	155.167	150.267	k€/MW	[3]
	VOM	0.553	0.554	0.554	0.554	€/MWh	[3]

	Economic Lifetime	25	25	25	25	years	[3]
Straw steam Subcritical Turbine condensing	Fuel efficiency	0.243	0.243	0.243	0.243	0-1	[3]
	Investment cost	3.212	3.072	2.926	2.780	M€/MW	[3]
	FOM	169.867	160.067	155.167	150.267	k€/MW	[3]
	VOM	2.434	2.439	2.439	2.439	€/MWh	[3]
	Economic Lifetime	25	25	25	25	years	[3]
Solar heating	Fuel efficiency	1	1	1	1	0-1	[3]
	Investment cost	0.415	0.368	0.350	0.333	M€/MW	[3]
	FOM	0.039	0.035	0.035	0.035	k€/MW	[3]
	VOM	0.103	0.147	0.159	0.172	€/MWh	[3]
	Economic Lifetime	27.5	30	30	30	years	[3]
Solar utility (PV)	Fuel efficiency	1	1	1	1	0-1	[3]
	Investment cost	0.612	0.459	0.396	0.345	M€/MW	[3]
	FOM	8.663	7.076	6.497	6.037	k€/MW	[3]
	VOM	0	0	0	0	€/MWh	[3]
	Economic Lifetime	35	40	40	40	years	[3]
Offshore wind near shore AC connection	Fuel efficiency	1	1	1	1	0-1	[26]
	Investment cost	1.956	1.634	1.511	1.474	M€/MW	[26]
	FOM	48.020	37.456	32.654	31.693	k€/MW	[26]
	VOM	4.802	3.740	3.285	3.123	€/MWh	[26]
	Economic Lifetime	27	30	30	30	years	[26]
Offshore wind far offshore AC connection	Fuel efficiency	1	1	1	1	0-1	[26]
	Investment cost	1.977	1.654	1.530	1.492	M€/MW	[26]
	FOM	48.020	37.456	32.654	31.693	k€/MW	[26]
	VOM	4.802	3.740	3.285	3.123	€/MWh	[26]
	Economic Lifetime	27	30	30	30	years	[26]
Offshore wind far offshore DC connection	Fuel efficiency	1	1	1	1	0-1	[26]
	Investment cost	2.048	1.719	1.592	1.551	M€/MW	[26]
	FOM	48.020	37.456	32.654	31.693	k€/MW	[26]
	VOM	4.802	3.740	3.285	3.123	€/MWh	[26]
	Economic Lifetime	27	30	30	30	years	[26]
Onshore wind	Fuel efficiency	1	1	1	1	0-1	[3]
	Investment cost	1.576	1.419	1.305	1.227	M€/MW	[3]
	FOM	21.658	19.933	18.799	18.230	k€/MW	[3]
	VOM	1.323	1.261	1.094	1.080	€/MWh	[3]
	Economic Lifetime	26.3	29	29	29	years	[3]
Woodchips heat only boiler	Fuel efficiency	1.017	1.025	1.025	1.025	0-1	[3]
	Investment cost	0.516	0.637	0.608	0.578	M€/MW	[3]
	FOM	22.115	30.576	29.645	28.714	k€/MW	[3]
	VOM	0.761	0.980	0.882	0.784	€/MWh	[3]
	Economic Lifetime	21.6667	25	25	25	years	[3]
Woodchips subcritical steam backpressure	Fuel efficiency	1.149	1.149	1.149	1.149	0-1	[3]
	Cb	0.280	0.280	0.280	0.280	-	[3]
	Investment cost	4.273	4.056	3.888	3.721	M€/MW	[3]
	FOM	156.800	150.267	145.367	140.467	k€/MW	[3]
	VOM	1.084	1.081	1.085	1.088	€/MWh	[3]
	Economic Lifetime	25	25	25	25	years	[3]
Woodchips Subcritical steam condensing	Fuel efficiency	0.247	0.247	0.247	0.247	0-1	[3]
	Investment cost	3.632	3.448	3.305	3.162	M€/MW	[3]
	FOM	156.800	150.267	145.367	140.467	k€/MW	[3]
	VOM	4.745	4.736	4.747	4.759	€/MWh	[3]
	Economic Lifetime	25	25	25	25	years	[3]
Woodpellets heat only boiler	Fuel efficiency	0.891	0.901	0.901	0.926	0-1	[3]
	Investment cost	0.686	0.736	0.713	0.690	M€/MW	[3]
	FOM	40.719	44.860	44.327	41.136	k€/MW	[3]
	VOM	0.250	0.250	0.225	0.200	€/MWh	[3]
	Economic Lifetime	22.5	22.5	22.5	22.5	years	[3]
Woodpellets subcritical steam backpressure	Fuel efficiency	0.993	0.993	0.993	0.993	0-1	[3]
	Cb	0.383	0.383	0.383	0.383	-	[3]
	Investment cost	3.523	3.352	3.209	3.066	M€/MW	[3]
	FOM	151.573	144.060	139.960	135.861	k€/MW	[3]
	VOM	0.506	0.505	0.380	0.507	€/MWh	[3]
	Economic Lifetime	25	25	25	25	years	[3]
Woodpellets subcritical steam condensing	Fuel efficiency	0.267	0.267	0.267	0.267	0-1	[3]
	Investment cost	2.919	2.773	2.654	2.534	M€/MW	[3]
	FOM	143.733	137.200	133.933	130.667	k€/MW	[3]
	VOM	2.047	2.044	2.047	2.051	€/MWh	[3]
	Economic Lifetime	25	25	25	25	years	[3]
Alkaline water electrolysis district heating	Fuel efficiency	0.656	0.672	0.707	0.741	0-1	[3]
	Investment cost	1.001	0.801	0.651	0.501	MEuro/MW	[21, 46]
	FOM	12.740	8.820	5.880	4.900	kEuro/MW	[3]
	VOM	-	-	-	-	Euro/MWh	[3]
	Economic lifetime	25	30	32	35	years	[3]
Alkaline water electrolysis	% to DH	6.100	6.840	9.540	15.770	%	[3]
	Fuel efficiency	0.656	0.672	0.707	0.742	0-1	[3]
	Investment cost	1.000	0.800	0.650	0.500	MEuro/MW	[46]
	FOM	12.740	8.820	5.880	4.900	kEuro/MW	[3]
	VOM	-	-	-	-	Euro/MWh	[3]
	Economic lifetime	25	30	32	35	years	[3]
Hydrogen to electricity (fuel cells)	Fuel efficiency	0.550	0.600	0.615	0.630	0-1	[3]
	Investment cost	1.470	0.784	0.637	0.490	MEuro/MW	[3]
	FOM	0.010	0.010	0.010	0.010	kEuro/MW	[3]
	VOM	9.800	3.920	3.430	2.940	Euro/MWh	[3]
	Economic lifetime	15	20	20	20	years	[3]
Hydrogen storage steel tank (140 bar)	Fuel efficiency	0.990	0.990	0.990	0.990	0-1	[3]
	Investment cost	0.056	0.044	0.026	0.021	MEuro/MW	[3]
	FOM	0.003	0.003	0.002	0.002	kEuro/MW	[3]
	VOM	-	-	-	-	Euro/MWh	[3]
	Economic lifetime	25	30	30	30	years	[3]
Hydrogen storage underground salt cavern	Fuel efficiency	0.990	0.990	0.990	0.990	0-1	[3]

	Investment cost	0.003	0.002	0.001	0.001	MEuro/MW	[3]
	FOM	0.059	0.039	0.029	0.024	Euro/MW	[3]
	VOM	0.000	0.000	0.000	0.000	Euro/MWh	[3]
	Economic lifetime	100	100	100	100	years	[3]
Hydrogen to biomethane + Direct air capture	Fuel efficiency	0.800	0.800	0.800	0.800	0-1	[61]
	Investment cost	1.250	1.250	1.250	1.250	MEuro/MW	[61]
	FOM	0.010	0.010	0.010	0.010	kEuro/MW	[61]
	VOM	-	-	-	-	Euro/MWh	[61]
	Economic lifetime	25	25	25	25	years	[61]
Steam methane reforming	Fuel efficiency	0.700	0.700	0.700	0.700	0-1	[3]
	Investment cost	0.571	0.571	0.571	0.571	MEuro/MW	[3]
	FOM	19.479	19.479	19.479	19.479	kEuro/MW	[3]
	VOM	-	-	-	-	Euro/MWh	[3]
	Economic lifetime	25	25	25	25	years	[3]
Steam methane reforming + CCS	Fuel efficiency	0.7	0.7	0.7	0.7	0-1	[3]
	Investment cost	1.191	1.191	1.191	1.191	MEuro/MW	[3]
	FOM	28.316	28.316	28.316	28.316	kEuro/MW	[3]
	VOM	-	-	-	-	Euro/MWh	[3]
	Economic lifetime	25	25	25	25	years	[3]
Biogas upgrading	Fuel efficiency	0.99	0.99	1	1	0-1	[3]
	Investment cost	0.419	0.373	0.355	0.336	MEuro/MW	[3]
	FOM	10.388	9.310	8.869	8.428	kEuro/MW	[3]
	VOM	-	-	-	-	Euro/MWh	[3]
	Economic lifetime	15	15	15	15	years	[3]
Biogas methanation	Fuel efficiency	1.679	1.679	1.679	1.679	0-1	[3]
	Investment cost	0.889	0.741	0.593	0.445	MEuro/MW	[3]
	FOM	35.562	29.635	23.708	17.781	kEuro/MW	[3]
	VOM	4.2336	3.528	2.8224	2.1168	Euro/MWh	[3]
	Economic lifetime	25	25	25	25	years	[3]

Supplementary References

[1] Hans, R. The Balmorel Model Structure (2018). URL http://www.balmorel.com/images/downloads/model/BMS303-20190311.pdf.

[2] Gea-Bermúdez, J. *et al.* The role of sector coupling in the green transition: A least-cost energy system development in Northern-central Europe towards 2050. *Applied Energy* **289**, 116685 (2021). URL https://linkinghub.elsevier.com/retrieve/pii/S0306261921002130.

[3] Danish Energy Agency (DEA). Technology Data (2023). URL https://ens.dk/en/our-services/projections-and-models/technology-data.

[4] DTU Computing Center. DTU Computing Center (2022). URL https://doi.org/10.48714/DTU.HPC.0001.

[5] Lambert, J., Hanel, A., Fendt, S. & Spliethoff, H. Evaluation of sector-coupled energy systems using different foresight horizons. *Renewable and Sustainable Energy Reviews* **184**, 113562 (2023). URL https://linkinghub.elsevier.com/retrieve/pii/S1364032123004197.

[6] Siala, K. *et al.* Which model features matter? An experimental approach to evaluate power market modeling choices. *Energy* **245**, 123301 (2022). URL https://linkinghub.elsevier.com/retrieve/pii/S0360544222002043.

[7] Babrowski, S., Heffels, T., Jochem, P. & Fichtner, W. Reducing computing time of energy system models by a myopic approach: A case study based on the PERSEUS-NET model. *Energy Systems* **5**, 65–83 (2014). URL http:

//link.springer.com/10.1007/s12667-013-0085-1.

[8] European Commission. Union methodology setting out detailed rules for the production of renewable liquid and gaseous transport fuels of non-biological originEuropean Commission (2023). URL https://eur-lex.europa.eu/eli/reg_del/2023/1184/oj.

[9] Rossum, R. *et al.* European Hydrogen Backbone - A EUROPEAN HYDROGEN INFRASTRUCTURE VISION COVERING 28 COUNTRIES. Tech. Rep. (2022). URL https://gasforclimate2050.eu/wp-content/uploads/2022/04/EHB-A-European-hydrogen-infrastructure-vision-covering-28-countries.pdf.

[10] IRENA. Global Hydrogen Trade to Meet the 1.5°C Climate Goal: Technology Review of Hydrogen Carriers (2022). URL https://www.irena.org/publications/2022/Apr/Global-hydrogen-trade-Part-II.

[11] Tareq, Z., Jaap, P., Matthias, S. & Jan, C. Facilitating hydrogen imports from non-EU countries. Tech. Rep. (2022). URL https://gasforclimate2050.eu/wp-content/uploads/2023/12/Facilitating-hydrogen-imports-from-non-EU-countries.pdf.

[12] Di Lullo, G. *et al.* Large-scale long-distance land-based hydrogen transportation systems: A comparative techno-economic and greenhouse gas emission assessment. *International Journal of Hydrogen Energy* **47**, 35293–35319 (2022). URL https://linkinghub.elsevier.com/retrieve/pii/S036031992203659X.

[13] Moritz, M., Schönfisch, M. & Schulte, S. Estimating global production and supply costs for green hydrogen and hydrogen-based green energy commodities. *International Journal of Hydrogen Energy* **48**, 9139–9154 (2023). URL https://linkinghub.elsevier.com/retrieve/pii/S0360319922057603.

[14] Franzmann, D. *et al.* Green hydrogen cost-potentials for global trade. *International Journal of Hydrogen Energy* **48**, 33062–33076 (2023). URL https://linkinghub.elsevier.com/retrieve/pii/S0360319923022401.

[15] Chen, P. S.-L. *et al.* A review on ports' readiness to facilitate international hydrogen trade. *International Journal of Hydrogen Energy* **48**, 17351–17369 (2023). URL https://linkinghub.elsevier.com/retrieve/pii/S0360319923004354.

[16] Lipiäinen, S., Lipiäinen, K., Ahola, A. & Vakkilainen, E. Use of existing gas infrastructure in European hydrogen economy. *International Journal of Hydrogen Energy* **48**, 31317–31329 (2023). URL https://linkinghub.elsevier.com/retrieve/pii/S0360319923021134.

[17] Topolski, K. *et al.* Hydrogen Blending into Natural Gas Pipeline Infrastructure: Review of the State of Technology. Tech. Rep. NREL/TP-5400-81704, 1893355, MainId:82477 (2022). URL https://www.osti.gov/servlets/purl/1893355/.

[18] IRENA. Global Hydrogen Trade to Meet the 1.5°C Climate Goal: Green Hydrogen Cost and Potential (2022).

[19] Hank, C. *et al.* Site-specific, comparative analysis for suitable Power-to-X pathways and products in developing and emerging countries. Tech. Rep. (2023). URL https://www.ise.fraunhofer.de/en/publications/studies/power-to-x-country-analyses.html.

[20] Gunkel, P. A., Koduvere, H., Kirkerud, J. G., Fausto, F. J. & Ravn, H. Modelling transmission systems in energy system analysis: A comparative study. *Journal of Environmental Management* **262**, 110289 (2020). URL https://linkinghub.elsevier.com/retrieve/pii/S0301479720302243.

[21] Gea-Bermúdez, J., Bramstoft, R., Koivisto, M., Kitzing, L. & Ramos, A. Going offshore or not: Where to generate hydrogen in future integrated energy systems? *Energy Policy* **174**, 113382 (2023). URL https://linkinghub.elsevier.com/retrieve/pii/S0301421522006012.

[22] Nordic Energy Research & International Energy Agency (IEA). Nordic Energy Technology Perspectives 2016. Tech. Rep. (2016). URL https://www.nordicenergy.org/wp-content/uploads/2016/04/Nordic-Energy-Technology-Perspectives-2016.pdf.

[23] Hofmann, F., Hampp, J., Neumann, F., Brown, T. & Hörsch, J. atlite: A Lightweight Python Package for Calculating Renewable Power Potentials and Time Series. *Journal of Open Source Software* **6**, 3294 (2021). URL https://joss.theoj.org/papers/10.21105/joss.03294.

[24] TRANSNET BW. ENERGY SYSTEM 2050 - Towards a decarbonised Europe. Tech. Rep. (2022). URL https://www.energysystem2050.net/content/TransnetBW-Study_EnergySystem2050.pdf?v2.

[25] Ruiz, P. *et al.* ENSPRESO - an open, EU-28 wide, transparent and coherent database of wind, solar and biomass energy potentials. *Energy Strategy Reviews* **26**, 100379 (2019). URL https://www.sciencedirect.com/science/article/pii/S2211467X19300720.

[26] Koivisto, M., Gea-Bermúdez, J. & Sørensen, P. North Sea offshore grid development: combined optimisation of grid and generation investments towards 2050. *IET Renewable Power Generation* **14**, 1259–1267 (2020). URL https://onlinelibrary.wiley.com/doi/10.1049/iet-rpg.2019.0693.

[27] Koivisto, M. *et al.* Using time series simulation tools for assessing the effects of variable renewable energy generation on power and energy systems. *WIREs Energy and Environment* **8** (2019). URL https://onlinelibrary.wiley.com/doi/10.1002/wene.329.

[28] Koivisto, M. J. & Murcia Leon, J. P. Pan-European wind and solar generation time series (PECD 2021 update) (2022). URL https://data.dtu.dk/collections/Pan-European_wind_and_solar_generation_time_series_PECD_2021_update_/5939581. Publisher: Technical University of Denmark.

[29] Murcia, J. P. *et al.* Validation of European-scale simulated wind speed and wind generation time series. *Applied Energy* **305**, 117794 (2022). URL https://linkinghub.elsevier.com/retrieve/pii/S0306261921011296.

[30] ENTSO-E. European Resource Adequacy Assessment (ERAA) 2021 (2021). URL https://www.entsoe.eu/outlooks/eraa/2021/eraa-downloads/.

[31] ENTSO-E & ENTSO-G. TYNDP 2022 Scenario Report (2022). URL https://2022.entsos-tyndp-scenarios.eu/download/.

[32] Tarvydas, D. The role of hydrogen in decarbonisation energy scenarios: views on 2030 and 2050. Tech. Rep., Publications Office, LU (2022). URL https://data.europa.eu/doi/10.2760/899528.

[33] Wang, A. *et al.* European Hydrogen Backbone - Analysing future demand, supply, and transport of hydrogen. Tech. Rep. (2021). URL https://gasforclimate2050.eu/wp-content/uploads/2021/06/EHB_Analysing-the-future-demand-supply-and-transport-of-hydrogen_June-2021.pdf.

[34] Pia Manz, T. F. Georeferenced industrial sites with fuel demand and excess heat potential (2018). URL https://zenodo.org/record/4687147. Version Number: 0.2.0 Type: dataset.

[35] Speth, D., Sauter, V., Plötz, P. & Signer, T. Synthetic European road freight transport flow data. *Data in Brief* **40**, 107786 (2022). URL https://linkinghub.elsevier.com/retrieve/pii/S235234092101060X.

[36] Speth, D., Sauter, V. & Plötz, P. Where to Charge Electric Trucks in Europe—Modelling a Charging Infrastructure Network 13 (2022).

[37] European Hydrogen Observatory. Scenarios for future hydrogen demand (2023). URL https://observatory.clean-hydrogen.europa.eu/tools-reports/scenarios-future-hydrogen-demand.

[38] Guidehouse. Five hydrogen supply corridors for Europe in 2030 (2022). URL https://ehb.eu/files/downloads/EHB-Supply-corridor-presentation-Full-version.pdf.

[39] SolarPower Europe. European Market Outlook for Solar Power 2022-2026. Tech. Rep. (2022). URL https://api.solarpowereurope.org/uploads/5222_SPE_EMO_2022_full_report_ver_03_1_319d70ca42.pdf?updated_at=

2022-12-19T08:21:34.541Z.

[40] European Commission. EU Reference Scenario 2020 (2020). URL https://energy.ec.europa.eu/data-and-analysis/energy-modelling/eu-reference-scenario-2020_en.

[41] Neumann, F., Zeyen, E., Victoria, M. & Brown, T. The potential role of a hydrogen network in Europe. *Joule* S2542435123002660 (2023). URL https://linkinghub.elsevier.com/retrieve/pii/S2542435123002660.

[42] Eurostat. Simplified energy balances (2023). URL https://ec.europa.eu/eurostat/databrowser/view/NRG_BAL_S__custom_4828709/default/table.

[43] Seck, G. S. *et al.* Hydrogen and the decarbonization of the energy system in europe in 2050: A detailed model-based analysis. *Renewable and Sustainable Energy Reviews* **167**, 112779 (2022). URL https://linkinghub.elsevier.com/retrieve/pii/S1364032122006633.

[44] Shirizadeh, B. *et al.* Towards a resilient and cost-competitive clean hydrogen economy: the future is green. *Energy & Environmental Science* 10.1039.D3EE02283H (2023). URL http://xlink.rsc.org/?DOI=D3EE02283H.

[45] Gawlick, J. & Hamacher, T. Impact of coupling the electricity and hydrogen sector in a zero-emission European energy system in 2050. *Energy Policy* **180**, 113646 (2023). URL https://linkinghub.elsevier.com/retrieve/pii/S0301421523002318.

[46] Odenweller, A., Ueckerdt, F., Nemet, G. F., Jensterle, M. & Luderer, G. Probabilistic feasibility space of scaling up green hydrogen supply. *Nature Energy* **7**, 854–865 (2022). URL https://www.nature.com/articles/s41560-022-01097-4.

[47] Zeyen, E., Victoria, M. & Brown, T. Endogenous learning for green hydrogen in a sector-coupled energy model for Europe. *Nature Communications* **14**, 3743 (2023). URL https://www.nature.com/articles/s41467-023-39397-2.

[48] International Renewable Energy Agency (IRENA). Global Hydrogen Review 2023. Tech. Rep. (2023). URL https://www.iea.org/reports/global-hydrogen-review-2023.

[49] Kountouris, I., Langer, L., Bramstoft, R., Münster, M. & Keles, D. Power-to-X in energy hubs: A Danish case study of renewable fuel production. *Energy Policy* **175**, 113439 (2023). URL https://linkinghub.elsevier.com/retrieve/pii/S0301421523000241.

[50] European Commission. REPowerEU: A plan to rapidly reduce dependence on Russian fossil fuels and fast forward the green transition (2022). URL https://ec.europa.eu/commission/presscorner/detail/en/IP_22_3131.

[51] Wetzel, M., Gils, H. C. & Bertsch, V. Green energy carriers and energy sovereignty in a climate neutral European energy system. *Renewable Energy* **210**, 591–603 (2023). URL https://linkinghub.elsevier.com/retrieve/pii/S0960148123004639.

[52] Jahanbakhsh, A., Louis Potapov-Crighton, A., Mosallanezhad, A., Tohidi Kaloorazi, N. & Maroto-Valer, M. M. Underground hydrogen storage: A UK perspective. *Renewable and Sustainable Energy Reviews* **189**, 114001 (2024). URL https://linkinghub.elsevier.com/retrieve/pii/S1364032123008596.

[53] Barison, E., Donda, F., Merson, B., Le Gallo, Y. & Réveillère, A. An Insight into Underground Hydrogen Storage in Italy. *Sustainability* **15**, 6886 (2023). URL https://www.mdpi.com/2071-1050/15/8/6886.

[54] Mattera, S. *et al.* First assessment of an area potentially suitable for underground hydrogen storage in Italy. *International Journal of Hydrogen Energy* **48**, 17940–17956 (2023). URL https://linkinghub.elsevier.com/retrieve/pii/S0360319923003841.

[55] Holz, F. *et al.* A 2050 perspective on the role for carbon capture and storage in the European power system and industry sector. *Energy Economics* **104**, 105631 (2021). URL https://linkinghub.elsevier.com/retrieve/pii/S0140988321004941.

[56] Emmerling, J. & Tavoni, M. Representing inequalities in integrated assessment modeling of climate change. *One Earth* **4**, 177–180 (2021). URL https://linkinghub.elsevier.com/retrieve/pii/S2590332221000592.

[57] Anthonsen, K. L. & Christensen, N. P. EU Geological C02 storage summary. Prepared by the Geological Survey of Denmark and Greenland for Clean Air Task Force [Revised, October 2021] URL https://data.geus.dk/gpub-landingpage/?id=34594. Publisher: [object Object].

[58] European Commission. Joint Research Centre. *Shaping the future CO2 transport network for Europe.* (Publications Office, LU, 2024). URL https://data.europa.eu/doi/10.2760/582433.

[59] Clean Air task force. Unlocking Europe's CO2 Storage Potential Analysis of Optimal CO2 Storage in Europe (2023). URL https://www.catf.us/resource/unlocking-europes-co2-storage-potential-analysis-optimal-co2-storage-europe/.

[60] International Energy Agency (IEA). World Energy Outlook 2022 (2022). URL https://www.iea.org/reports/world-energy-outlook-2022.

[61] Brown, T., Schlachtberger, D., Kies, A., Schramm, S. & Greiner, M. Synergies of sector coupling and transmission reinforcement in a cost-optimised, highly renewable European energy system. *Energy* **160**, 720–739 (2018). URL https://linkinghub.elsevier.com/retrieve/pii/S036054421831288X.
